# Targeting carnitine palmitoyl transferase 1A (CPT1A) induces ferroptosis and synergizes with immunotherapy in lung cancer

**DOI:** 10.1038/s41392-024-01772-w

**Published:** 2024-03-07

**Authors:** Lei Ma, Chong Chen, Chunxing Zhao, Tong Li, Lingyu Ma, Jiayu Jiang, Zhaojun Duan, Qin Si, Tsung-Hsien Chuang, Rong Xiang, Yunping Luo

**Affiliations:** 1 Department of Immunology, Institute of Basic Medical Sciences, Chinese Academy of Medical Sciences; School of Basic Medicine, Peking Union Medical College, Beijing, 100005 China; 2 Collaborative Innovation Center for Biotherapy, Institute of Basic Medical Sciences, Chinese Academy of Medical Sciences; School of Basic Medicine, Peking Union Medical College, Beijing, 100005 China; 3 Department of Lung Cancer Surgery, Tianjin Medical University General Hospital, Tianjin, 300052 China; 4 Immunology Research Center, National Health Research Institutes, Zhunan, Miaoli, Taiwan, ROC; 5 Department of Immunology, Nankai University, Tianjin, 300071 China

**Keywords:** Cancer metabolism, Cancer stem cells, Cancer microenvironment, Cancer therapy, Tumour immunology

## Abstract

Despite the successful application of immune checkpoint therapy, no response or recurrence is typical in lung cancer. Cancer stem cells (CSCs) have been identified as a crucial player in immunotherapy-related resistance. Ferroptosis, a form of cell death driven by iron-dependent lipid peroxidation, is highly regulated by cellular metabolism remolding and has been shown to have synergistic effects when combined with immunotherapy. Metabolic adaption of CSCs drives tumor resistance, yet the mechanisms of their ferroptosis defense in tumor immune evasion remain elusive. Here, through metabolomics, transcriptomics, a lung epithelial-specific *Cpt1a*-knockout mouse model, and clinical analysis, we demonstrate that CPT1A, a key rate-limiting enzyme of fatty acid oxidation, acts with L-carnitine, derived from tumor-associated macrophages to drive ferroptosis-resistance and CD8^+^ T cells inactivation in lung cancer. Mechanistically, CPT1A restrains ubiquitination and degradation of c-Myc, while c-Myc transcriptionally activates *CPT1A* expression. The CPT1A/c-Myc positive feedback loop further enhances the cellular antioxidant capacity by activating the NRF2/GPX4 system and reduces the amount of phospholipid polyunsaturated fatty acids through ACSL4 downregulating, thereby suppressing ferroptosis in CSCs. Significantly, targeting CPT1A enhances immune checkpoint blockade-induced anti-tumor immunity and tumoral ferroptosis in tumor-bearing mice. The results illustrate the potential of a mechanism-guided therapeutic strategy by targeting a metabolic vulnerability in the ferroptosis of CSCs to improve the efficacy of lung cancer immunotherapy.

## Introduction

Lung cancer is the leading cause of cancer-related deaths, with non-small cell lung cancer (NSCLC) accounting for 80% of cases.^1^ Despite the availability of molecular targeted therapies and immunotherapy for patients with lung cancer, recurrence and disease progression due to drug resistance are common.^2^ Cancer stem cells (CSCs), which are pivotal drivers of tumor initiation, resistance, and metastasis, play a critical role in tumor immune evasion and immunotherapy tolerance.^3–5^ CSCs possess the potential for self-renewal and differentiation, which fuels and sustains tumor growth at low cell numbers, and they must first overcome the formidable barrier of immune surveillance. It is speculated that some CSCs evade detection and eradication by immune cells to escape antitumor immunity; however, the specific mechanisms behind this phenomenon remain largely unknown.

Ferroptosis, a type of cell death driven by iron-dependent lipid peroxidation, has been implicated in various biological processes including development, aging, immunity, and cancer.^6,7^ The convergence of three significant areas of research—metabolism, reactive oxygen species (ROS) biology, and iron regulation—has laid the groundwork for understanding ferroptosis.^6^ Recent findings have shed light on the metabolic plasticity of CSCs and have provided intriguing insights into how metabolic rewiring plays a crucial role in CSCs’ resistance to ferroptosis.^8,9^ In certain instances, this metabolic reprogramming has been associated with an acquired sensitivity to ferroptosis, presenting new possibilities for treating therapy-resistant tumors. Moreover, CSCs often express elevated levels of antioxidant proteins to reduce ROS levels and maintain redox homeostasis, which potentially confers resistance to ferroptosis.^8–11^ Furthermore, cancer cells undergoing ferroptosis have been shown to release various damage-associated molecular patterns (DAMPs).^12,13^ Hence, ferroptosis could potentially serve as a form of immunogenic cell death, and augmenting CSCs’ susceptibility to ferroptosis may boost the effectiveness of immunotherapy in lung cancer treatment. Mounting evidence indicates that fatty acid oxidation (FAO) metabolism is essential for CSCs survival and plays a critical role in ferroptosis.^14,15^ Carnitine palmitoyl transferase 1A (CPT1A) is a crucial enzyme in FAO that regulates the transport of long-chain fatty acids into the mitochondria for fatty acid β-oxidation. CPT1A has been identified as an important driver of tumor progression and is implicated in various aspects of cancer development and metastasis.^16^ Pharmacological inhibition of fatty acid oxidation using CPT1 inhibitors consistently sensitized tumor cells to chemotherapy or immunotherapy-induced tumor cell death.^17,18^ Metabolic reprogramming, driven by mutations in cancer genes and changes in cellular signaling controlled by key oncogenes like c-Myc and mutant RAS, is a crucial factor in lung cancer progression. Oncogenic c-Myc plays a significant role in metabolic remodeling and orchestrates an antioxidant response that is essential for maintaining redox balance and promoting tumor cell survival. However, the potential regulatory role of the interplay between CPT1A and c-Myc in modulating the resistance of CSCs to ferroptosis and immune evasion remains largely unexplored.

Metabolic reprogramming in tumor cells is often shaped by the environmental factors within the tumor microenvironment (TME), which encompass gradients of nutrient and oxygen levels, tissue vascularization, heterocellular interactions, and systemic metabolism.^19^ Extracellular metabolites serve not only as a source of energy supply but also as communication signals between different cellular compartments.^20^ Recently, there has been emerging evidence indicating that abnormal metabolites in the TME play a role in modulating tumoral ferroptosis and immune evasion.^21,22^ L-carnitine, an amino acid-like substance primarily obtained from food and present in approximately 75% of humans, serves as a carrier for the mitochondrial translocation of long-chain fatty acids in the FAO process.^23^ Abnormally high levels of L-carnitine have been observed in patients with lung or colon cancers.^24^ Immune cells within the TME are known to promote tumor growth, metastasis, and resistance to cancer therapy, underscoring the clinical potential of TME-targeted therapies. Tumor-associated macrophages (TAMs), possessing anti-inflammatory properties, play a pivotal role in maintaining CSCs, promoting metastasis, and conferring therapeutic resistance.^25^ The phenotypes of TAMs are also influenced by cell-intrinsic metabolism, with significant functional implications for the tumor microenvironment. While the emerging role of TAMs in preventing tumoral ferroptosis has been recently proposed,^26^ the specific metabolic interplay underlying this phenomenon remains largely unknown. Given the supportive effect of TAMs on the maintenance of CSCs and the contribution of CTLs to initiating tumoral ferroptosis, targeting the crosstalk between TAMs and ferroptosis may hold promise for reversing immunotherapeutic resistance in lung cancer.

This study delved into the role of CPT1A in conferring resistance to ferroptosis and immune evasion in lung cancer stem cells (LCSCs). Our investigations revealed a novel mechanism in which the CPT1A/c-Myc positive feedback loop, supported by L-carnitine derived from TAMs, hinders CD8^+^ T cell-induced tumor ferroptosis. The CPT1A/c-Myc loop predominantly suppresses tumoral ferroptosis through the NRF2/GPX4 and ACSL4/PUFA-PLs pathways, and targeted inhibition of CPT1A disrupts this detrimental cycle, thereby enhancing the efficacy of immune checkpoint therapy in lung cancer.

## Results

### CPT1A is indispensable for tumor progression and LCSCs maintenance in lung cancer

**Fig. 1:**
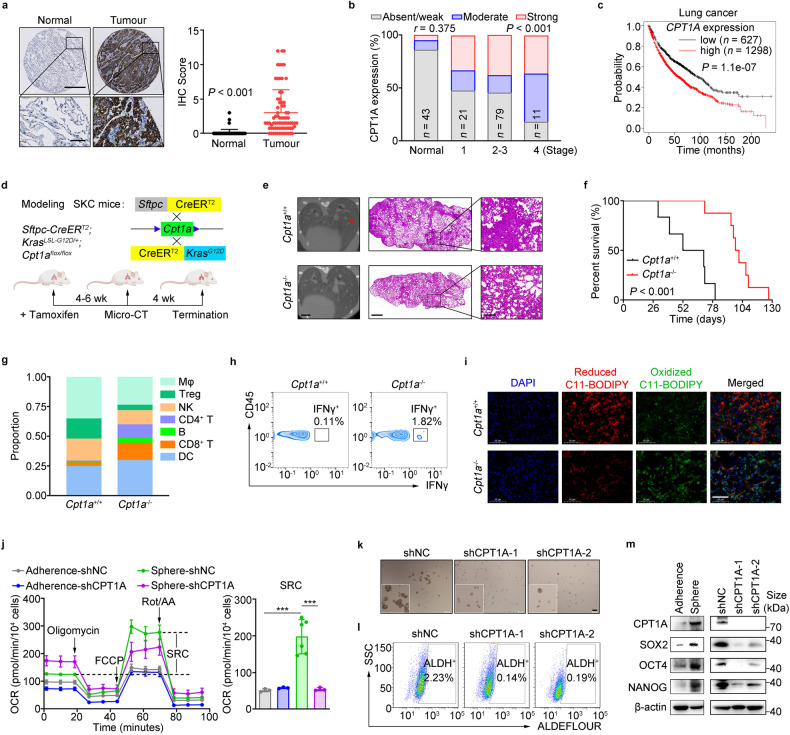
CPT1A is indispensable for tumor progression and LCSCs maintenance in lung cancer. **a**–**c** Relevance of CPT1A expression with the tumor progression in NSCLC patients. Representative IHC staining for CPT1A in the normal or cancerous lung tissues from NSCLC patients (left) and IHC score of CPT1A were depicted on the right. Scale bars, 500 μm (up); 50 μm (down) (insets) (**a**). Correlation of CPT1A level with the clinical stages of NSCLC patients (**b**) and overall survival (OS) of NSCLC patients with lung cancer stratified according to the expression of CPT1A in the tumor (**c**). **d**–**i** Effects of Cpt1a on tumor progression in the transgenic mouse. Construction strategy for the transgenic SKC (*Sftpc*-CreER^T2^; *Kras*^G12D^; *Cpt1a*^flox/flox^) mice and the experimental scheme (**d**). Representative micro-CT image (left) and H&E staining (right) of the lung tissue from SKC or control mice. Scale bars, 2 mm (micro-CT); 1 mm (H&E, left); 5 mm (H&E, right) (insets) (**e**). Overall survival of SKC or control mice (**f**). Percentages of distinct types of immune cells (**g**) and IFNγ^+^ cells gated on CD45^+^ cells in the lung tissues from SKC or control mice (**h**). Representative images of C11-BODIPY staining in the lung tissue from SKC or control mice. Scale bars, 50 μm (insets) (**i**). **j** Spare respiratory capacity in adherent or spheroid cells of LLC-shNC/shCPT1A cells measured by using Seahorse XF Pro analyzer with mitochondrial stress test. Data represent the mean ± s.d.; *n* = 3-6 samples. **k** Representative tumor-sphere images of H1299-shNC/shCPT1A cells cultured with sphere-forming medium. Scale bars, 200 μm (insets)；zoomed images (left bottom, 2.5 times amplification). **l** Percentages of ALDH^+^ cells in H1299-shNC/shCPT1A cells determined by flow cytometry. **m**, Representative western blot for CPT1A, SOX2, OCT4 and NANOG in adherent, spheroid or shNC/shCPT1A cells of H1299 cells. *P < 0.05, **P < 0.01, ***P < 0.001; ns: not significant. The statistical analysis was performed using a two-tailed Student’s *t*-test or Pearson’s correlation test

FAO metabolism played pivotal roles in tumor initiation, metastasis, or resistance,^14,15,27^ and we proposed that CPT1, the rate-limiting enzyme in FAO, was essential for NCSLC progression. Among the three isoforms of the *CPT1* gene (*CPT1A*, *CPT1B*, and *CPT1C*), amplification of the *CPT1A* gene was found to be the most frequent in patients with NSCLC. Additionally, the expression levels of *Cpt1a* transcripts were notably higher in LLC cells (Supplementary Fig. 1a, b). Importantly, the levels of CPT1A were significantly elevated in tumor tissues compared to adjacent normal tissues in lung cancer patients. Furthermore, higher expression levels of CPT1A were associated with more severe pathological grade and shorter survival time in these patients (Fig. 1a–c and Supplementary Fig. 1c).

To rigorously investigate the function of the *Cpt1a* gene in tumorigenesis, we developed a transgenic mouse model with spontaneous pulmonary carcinoma and targeted knockout of *Cpt1a* specifically in pulmonary epithelial cells (*Sftpc-*CreER^T2^; *Kras*^LSL-G12D^; *Cpt1a*^flox/flox^), referred to as SKC mice (Fig. 1d). We observed a significant decrease in the number and area of tumor nodules in the *Cpt1a*-knockout mice (*Cpt1a*^-/-^) compared to the control mice (*Cpt1a*^*+/+*^), also a prolonged survival time (Fig. 1e, f and Supplementary Fig. 1d). Notably, the immunosuppressive state of lung tissue was remodeled in *Cpt1a*^-/-^ mice, particularly showing an increased infiltration and activation of CD8^+^ T cells (Fig. 1g, h). Immunogenic tumor ferroptosis, which was induced by CD8^+^ T cell-derived IFNγ during the tumor regression,^28^ was also dramatically enhanced in *Cpt1a*^-/-^ mice (Fig. 1i). Furthermore, the tumor-promoting effect of CPT1A was confirmed in a syngeneic mouse model inoculated with LLC-shNC or LLC-shCPT1A cells (Supplementary Fig. 1e–i).

Enhanced FAO metabolism was observed in CSCs,^15,29^ and our data indicated that spheroid cells showed an increased spare respiration capacity (SRC), which disappeared upon knockdown of CPT1A (Fig. 1j). Additionally, the expression of *CPT1A* along with stemness genes was all increased in tumorsphere, ALDH^+^ or CD44^+^ cells; however, decreased number of spheres or ALDH^+^ cells and reduced expression of stemness genes were shown in H1299-shCPT1A cells (Fig. 1k–m and Supplementary Fig. 1j). Furthermore, our results showed that etomoxir (ETO), a specific small molecule inhibitor targeting CPT1A, displayed a robust inhibitory effect on stemness phenotypes (Supplementary Fig. 1k–m). Overall, our findings strongly support the notion that CPT1A serves as a powerful driver for both the initiation and progression of lung cancer, with a profound impact on cancer stemness.

### CPT1A is an essential driver for ferroptosis resistance in LCSCs

**Fig. 2:**
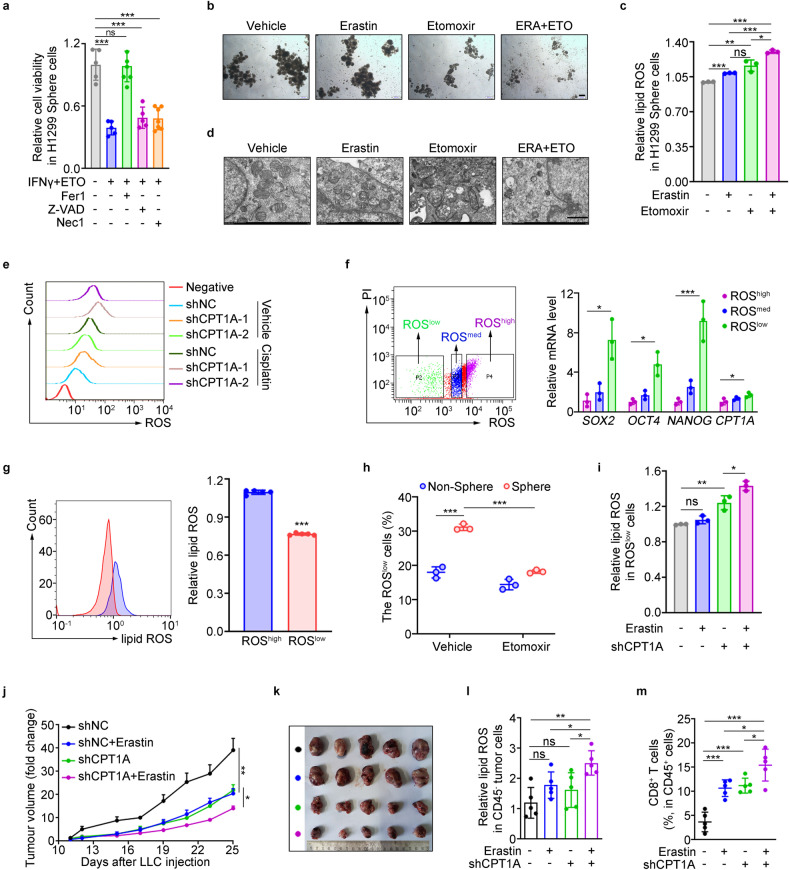
CPT1A is an essential driver for ferroptosis resistance in LCSCs. **a** Cell viability of H1299 sphere cells treated with IFNγ (100 μM) and etomoxir (100 μM) in the presence of ferrostatin-1 (Fer1, 2 mM), necostatin-1 (Nec1, 1 mM), or z-VAD-FMK (z-VAD, 10 mM) for 2 days detected by colorimetry. Data represent the mean ± s.d.; *n* = 5–7 samples. **b** Representative tumor-sphere images of LLC cells treated with etomoxir (100 μM) for 2 days and then erastin (2 μM) for 6 h. Scale bars, 200 μm (insets). **c** Lipid ROS levels in H1299 sphere cells treated with etomoxir (100 μM) for 2 days and then erastin (2 μM) for 6 h determined by flow cytometry. Data represent the mean ± s.d.; *n* = 3 samples. **d** Representative images of transmission electron microscope analyzed in H1299-shNC/shCPT1A cells treated with etomoxir (100 μM) for 2 days and additional erastin (2 μM) for 6 h. Scale bar, 1 μm (insets). **e** ROS levels in H1299-shNC/shCPT1A cells treated with DMSO or cisplain for 2 days. **f** Quantification of mRNA for *SOX2*, *OCT4*, *NANOG* and *CPT1A* in ROS^high^, ROS^medium^ or ROS^low^ cells isolated by flow cytometry, respectively. **g** Lipid ROS levels in ROS^high^ or ROS^low^ cells of H1299 cells determined by flow cytometry. Data represent the mean ± s.d.; *n* = 5 samples. **h** Percentage of ROS^low^ cells in sphere or non-sphere cells of LLC cells treated with etomoxir (100 μM) or not for 2 days determined by flow cytometry. Data represent the mean ± s.d.; *n* = 3 samples. **i** Lipid ROS levels in ROS^low^ cells of LLC-shNC/shCPT1A cells treated with erastin (2 μM) or not for 6 h determined by flow cytometry. Data represent the mean ± s.d.; *n* = 3 samples. Effects of erastin (IP, 20 mg/kg) on tumor progression in tumor-bearing mice inoculated with LLC-shNC/shCPT1A cells. Tumor growth curve (data represent the mean ± s.e.m.; *n* = 5 samples) (**j**). Photograph of the mouse tumor (**k**). Lipid ROS levels of CD45^-^ cells (**l**) and percentage of CD8^+^ T cells in the tumor (**m**). Data represent the mean ± s.d.; *n* = 5 samples. *P < 0.05, **P < 0.01, ***P < 0.001; ns: not significant. The statistical analysis was performed using a two-tailed Student’s *t*-test or Pearson’s correlation test

Given the role of CPT1A in cancer stemness maintenance, we sought to investigate whether CPT1A contributes to therapeutic resistance in LCSCs. Initially, our findings revealed that H1299-shCPT1A cells treated with IFNγ or cisplatin exhibited a significantly increased rate of cell death, although the number of apoptotic cells remained unaltered (Supplementary Fig. 2a, b). This observation suggests that the pro-survival mechanism mediated by CPT1A may not be dependent on the apoptosis pathway in lung cancer cells. To ascertain whether the induced cellular death resulting from this treatment was attributable to ferroptosis, we added various cell death inhibitors, including ferrostatin-1 (Fer1), a specific ferroptosis inhibitor, necrostatin-1 (Nec1), a necrosis inhibitor, and Z-VAD-fmk (Z-VAD), an apoptosis inhibitor, to the cancer cells treated with IFNγ and etomoxir. Our results demonstrated that Fer1 notably rescued cellular death, particularly in tumor spheres (Fig. 2a and Supplementary Fig. 2c). Furthermore, the combination of etomoxir with erastin or IFNγ significantly reduced the number of spheres and induced lipid-ROS (ferroptosis indicator) levels in tumor cells (Fig. 2b, c and Supplementary Fig. 2d, e). Additionally, the combined treatment led to an increased abundance of malondialdehyde (MDA), an indicator of lipid peroxidation, and the morphological characteristics of ferroptosis in H1299 cells, including rounded and shrunken mitochondria and the disappearance of mitochondrial inner ridges (Fig. 2d and Supplementary Fig. 2f).

Furthermore, considering the pivotal role of CPT1A in oxidative metabolism and cellular redox state, we proceeded to assess the level of ROS, which are known to influence cell survival.^30^ Our findings demonstrated that the total ROS level was elevated in H1299-shCPT1A cells treated with cisplatin, compared to untreated cells (Fig. 2e and Supplementary Fig. 2g, h). Notably, a previous study indicated that leukemia stem cells are characterized by relatively low levels of ROS, referred to as “ROS^low^" cells.^31^ Given the close relationship between ferroptosis and cellular ROS levels, we aimed to investigate whether the ROS^low^ population in lung cancer cells contributes to the enrichment of CSCs and confers resistance to ferroptosis. Firstly, we isolated three distinct subpopulations based on their disparate ROS levels and observed significant upregulation of stemness genes, including *CPT1A*, in the ROS^low^ or spheroid cells. Additionally, the level of ferroptosis was substantially attenuated in these cells (Fig. 2f, g and Supplementary Fig. 2h). Secondly, we observed a higher enrichment of ROS^low^ cells in spheres compared to non-spheres, and the introduction of etomoxir significantly reduced the proportion of ROS^low^ cells in the spheroid population (Fig. 2h). Thirdly, ferroptosis was prominently induced in the ROS^low^ cells of LLC-shCPT1A cells treated with erastin (Fig. 2i), and the population of ROS^low^ cells was significantly enriched in H1299 cells treated with cisplatin alone but was rapidly eliminated when combined with shCPT1A or etomoxir (Supplementary Fig. 2i). Importantly, erastin exhibited enhanced anti-tumor effects in the case of CPT1A-deficiency in a subcutaneous tumor-bearing mouse model (Fig. 2j, k), resulting in a higher degree of tumoral ferroptosis and infiltration of CD8 ^+^ T cells (Fig. 2l, m). Collectively, these findings provide compelling evidence that CPT1A promotes ferroptosis resistance to support the survival of LCSCs and drive the progression of lung cancer.

### CPT1A preserves the antioxidant state and restricts phospholipid polyunsaturated fatty acid production in lung cancer cells

**Fig. 3:**
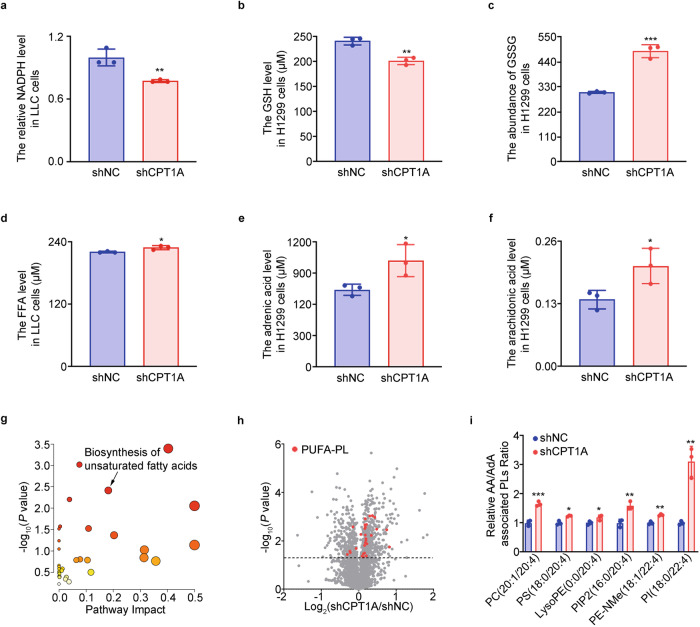
CPT1A preserves the antioxidant state and restricts phospholipid polyunsaturated fatty acid production in lung cancer cells. **a**–**c** Analysis of cellular redox state in H1299-shNC/shCPT1A cells. Levels of NADPH (**a**), GSH (**b**) and GSSG (**c**) calculated by colorimetry or LC-MS. Data represent the mean ± s.d.; *n* = 3 samples. **d**–**f** Analysis of fatty acid in H1299-shNC/shCPT1A cells. Levels of free fatty acid (**d**), adrenic acid (**e**) and arachidomic acid (**f**) calculated by LC-MS. Data represent the mean ± s.d.; *n* = 3 samples. **g**–**i** Analysis of PUFA-PLs in H1299-shNC/shCPT1A cells. KEGG analysis of the untargeted lipidomic data (**g**), volcano plots of lipid species (**h**) and levels of polyunsaturated fatty acids with adrenic acid or arachidomic acid branches (**i**) detected by LC-MS. Data represent the mean ± s.d.; *n* = 3 samples. *P < 0.05, **P < 0.01, ***P < 0.001; ns: not significant. The statistical analysis was performed using a two-tailed Student’s *t*-test

To understand the influence of CPT1A on lipid substrates and oxidants in driving ferroptosis, it is crucial to examine the oxidation of specific lipids. Our findings shed light on this aspect. We observed that the levels of cellular antioxidants, NADPH and reduced glutathione (GSH), were significantly reduced in CPT1A-deficient lung cancer cells. Conversely, the level of oxidized glutathione (GSSG) was increased (Fig. 3a–c and Supplementary Fig. 3a). These results suggest that CPT1A plays a critical role as an antioxidant factor in preventing oxidative damage in lung cancer cells. Furthermore, we investigated the impact of CPT1A on free fatty acid levels, particularly adrenic acid (AdA) and arachidonic acid (AA). Our data revealed elevated levels of these free fatty acids in CPT1A-deficient lung cancer cells (Fig. 3d–f and Supplementary Fig. 3a). Through untargeted lipidomics analysis, we identified significant alterations in the unsaturated fatty acid synthesis pathway and observed increased levels of phospholipid polyunsaturated fatty acids (PUFA-PLs), specifically those containing AA and AdA branches, in H1299-shCPT1A cells (Fig. 3g–i). These findings propose two independent mechanisms through which CPT1A tightly controls the occurrence of ferroptosis in LCSCs: regulation of the cellular redox state and accumulation of PUFA-PLs.

### CPT1A directly sustains the protein stability of c-Myc to promote ferroptosis resistance in lung cancer cells

**Fig. 4:**
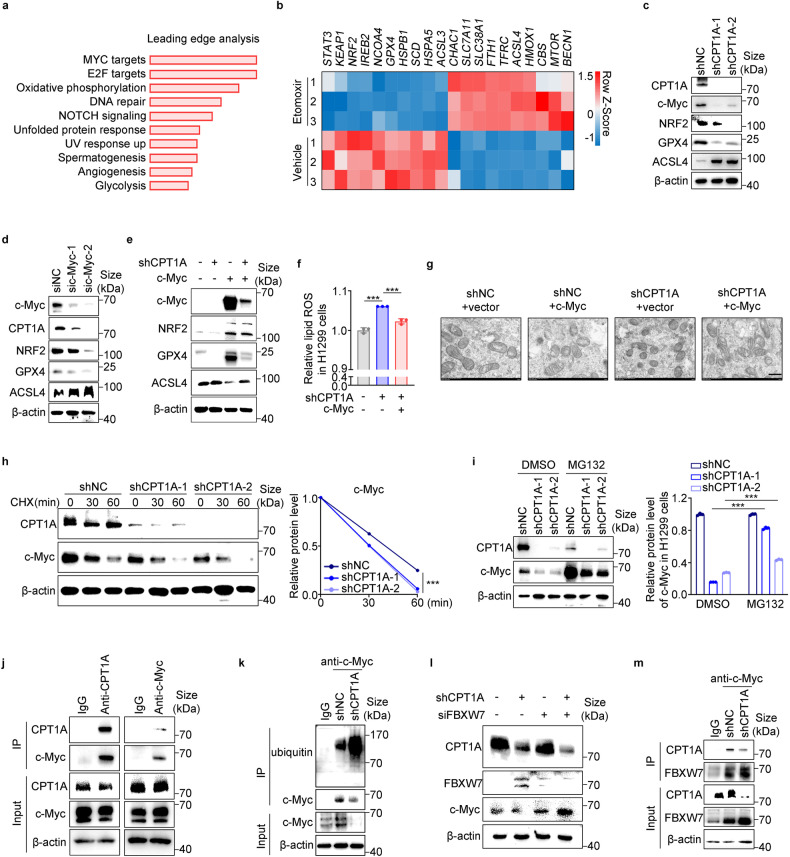
CPT1A directly sustains the protein stability of c-Myc to promote ferroptosis resistance in lung cancer cells. Analysis of RNA-seq data of H1299 cells treated with etomoxir (100 μM) or not for 2 days Leading-edge analysis for the GSEA (**a**). **b** Heatmap of RNA-seq data depicting the crucial changed genes regulating ferroptosis (Q < 0.05) (**b**). **c** Representative western blot for CPT1A, c-Myc, NRF2, GPX4 and ACSL4 in H1299-shNC/shCPT1A cells. **d** Representative western blot for c-Myc, CPT1A, NRF2, GPX4 and ACSL4 in H1299 cells transfected with sic-Myc or control siRNA. **e** Representative western blot for c-Myc, NRF2, GPX4 and ACSL4 in H1299-shNC/shCPT1A cells transfected with c-Myc or control plasmid. **f** Lipid ROS levels in H1299-shNC/shCPT1A cells transfected with c-Myc or control plasmid determined by flow cytometry. Data represent the mean ± s.d.; *n* = 3 samples. **g** Morphology of mitochondria detected by transmission electron microscope in H1299-shNC/shCPT1A cells transfected with c-Myc or control plasmid. Scale bar, 500 nm (insets). **h** Representative western blot for CPT1A and c-Myc in H1299-shNC/shCPT1A cells treated with cycloheximide (100 μg/mL) (left) and quantified by ImageJ software (right). **i** Representative western blot for CPT1A and c-Myc in H1299-shNC/shCPT1A cells treated with MG132 (10 mM) (left) and quantified by ImageJ software (right). **j** Co-immunoprecipitation with CPT1A (left) or c-Myc antibody (right) and representative western blot for CPT1A and c-Myc in H1299 cells. **k** Co-immunoprecipitation with c-Myc antibody and representative western blot for ubiquitin and c-Myc in H1299-shNC/shCPT1A cells. **l** Representative western blot for CPT1A, FBXW7 and c-Myc in H1299-shNC/shCPT1A cells transfected with siFBXW7 or control siRNA. **m** Co-immunoprecipitation with c-Myc antibody and representative western blot for CPT1A and FBXW7 in H1299-shNC/shCPT1A cells. **P < 0.01, ***P < 0.001; ns: not significant. The statistical analysis was performed using a two-tailed Student’s *t* test or Pearson’s correlation test

To further elucidate the mechanism by which CPT1A regulates ferroptosis in LCSCs, we conducted transcriptomic analysis of H1299 cells treated with etomoxir. Our findings revealed that the most significant change occurred in c-Myc targets, as indicated by gene set enrichment analysis (GSEA) (Fig. 4a). Additionally, through our analysis, we identified *c-Myc*, *NRF2*, *GPX4*, and *ACSL4* as the top ferroptosis-related genes regulated by CPT1A in lung cancer cells (Fig. 4b, c and Supplementary Fig. 3b, c).

Next, our investigation aimed to elucidate how CPT1A regulates c-Myc expression in lung cancer cells. Our results indicated that CPT1A does not directly regulate c-Myc transcription (Supplementary Fig. 3c). However, we observed that knockdown of CPT1A significantly compromised the stability of the c-Myc protein in cells treated with cycloheximide (CHX) (Fig. 4h). Given that the ubiquitin-proteasome system governs the stability of c-Myc, we treated the cells with MG132, a proteasome inhibitor. As anticipated, MG132 treatment led to a substantial increase in c-Myc protein levels, and the reduced amount of c-Myc protein in CPT1A-deficient cells was partially restored after MG132 treatment (Fig. 4i).

Moreover, cellular immunofluorescence analysis revealed colocalization of the CPT1A and c-Myc proteins in the cytoplasm. Additionally, each protein could be detected in the complex pulled down by the antibody of the other protein in several cell lines (Fig. 4j and Supplementary Fig. 4a–c). Notably, when CPT1A was knocked down, it resulted in enhanced ubiquitination of the c-Myc protein and a decreased binding of c-Myc protein to CPT1A (Fig. 4k and Supplementary Fig. 4d). We also investigated the expression of critical regulators for c-Myc ubiquitination reported previously.^31^ Our findings revealed a significant increase in the mRNA level of *FBXW7* (a ubiquitin ligase), as well as its protein level, in CPT1A-deficient lung cancer cells (Fig. 4l and Supplementary Fig. 4e). Furthermore, when c-Myc expression was up-regulated in CPT1A-deficient cells due to siFBXW7, the protein level of FBXW7 binding with c-Myc was also increased (Fig. 4l, m). These results unveil a novel mechanism in which CPT1A stabilizes the c-Myc protein by partially suppressing its ubiquitination and degradation through competitive inhibition of FBXW7 binding to c-Myc in lung cancer cells.

### c-Myc transcriptionally activates CPT1A to form a mutually reinforcing intermolecular loop in lung cancer cells

**Fig. 5:**
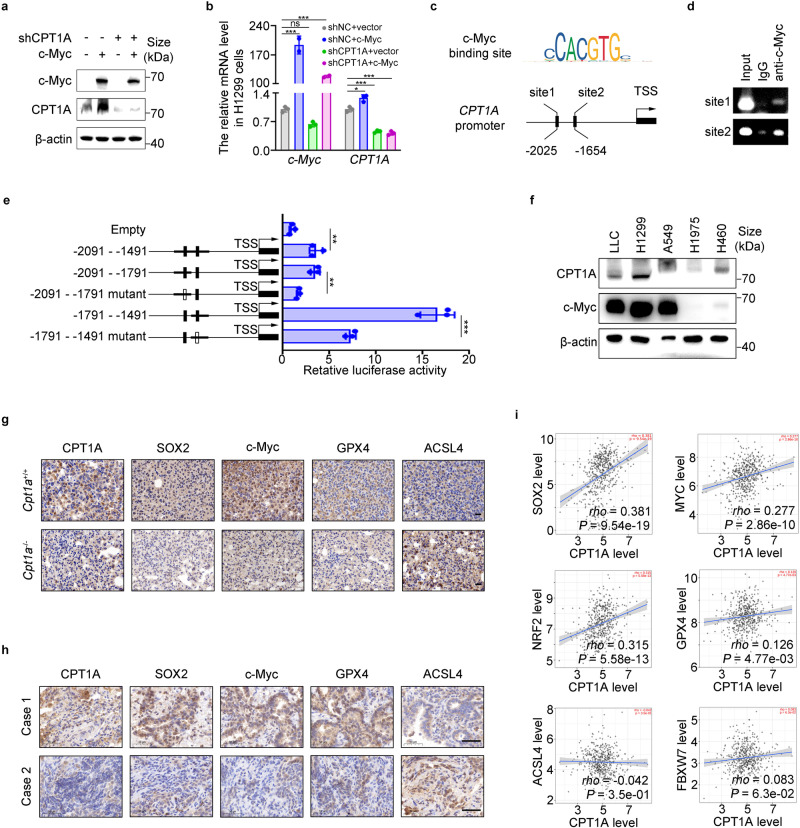
c-Myc transcriptionally activates *CPT1A* to form a mutually reinforcing intermolecular loop in lung cancer cells. Analysis of CPT1A expression in H1299-shNC/shCPT1A cells transfected with c-Myc or control plasmid. Quantification of mRNA (**a**) and representative western blot for c-Myc and CPT1A (**b**). Data represent the mean ± s.d.; *n* = 3 samples. **c** The DNA sequence of c-Myc binding motif and the binding sites of c-Myc in the promotor of *CPT1A* gene predicted by PROMO website. **d** Chromatin immunoprecipitation with c-Myc antibody and RT-PCR for c-Myc binding sites. **e** A dual-luciferase reporter assay to investigate the transcriptional activity of the c-Myc binding sites in H1299 cells transfected with plasmids containing wild type or mutant binding site. Data represent the mean ± s.d.; *n* = 3 samples. **f** Representative western blot for CPT1A and c-Myc in distinct lung cancer cell lines. **g** Representative IHC staining for CPT1A, SOX2, c-Myc, GPX4 and ACSL4 in the tumor of *Cpt1a*^+/+^ or *Cpt1a*^-/-^ transgenic mice. Scale bar: 20 μm (insets). **h** Representative IHC staining for CPT1A, SOX2, c-Myc, GPX4 and ACSL4 in the tumor of NSCLC patients. Scale bar: 50 μm (insets). **i** Correlation analysis between *CPT1A* and *SOX2/c-Myc/NRF2/GPX4/ACSL4/FBXW7* transcripts by using TCGA dataset. *P < 0.05, **P < 0.01, ***P < 0.001; ns: not significant. The statistical analysis was performed using a two-tailed Student’s *t*-test or Pearson’s correlation test or log-rank (Mantel–Cox) test

Our investigation revealed a tight regulation of CPT1A expression by c-Myc (Fig. 5a, b), indicating a positive regulatory effect between them. We hypothesized that c-Myc functioned as a transcriptional activator for the *CPT1A* gene. Using PROMO, we identified two binding sites of c-Myc in the promoter region of the *CPT1A* gene and confirmed their binding through chromatin immunoprecipitation (ChIP) assay (Fig. 5c, d). A dual-luciferase reporter assay revealed that site two displayed a more robust transcriptional activity than site one (Fig. 5e). These findings demonstrated that c-Myc directly activated CPT1A transcription by binding to its promoter.

We also analyzed the correlation between CPT1A and c-Myc in various lung cancer cell lines and found that CPT1A levels were increased in cells abundant in c-Myc (Fig. 5f). Furthermore, IHC staining of tumor tissues in NSCLC patients or *Cpt1a*-knockout mouse revealed that the protein level of CPT1A positively correlated with SOX2, c-Myc, and GPX4, but negatively correlated with ACSL4 (Fig. 5g, h and Supplementary Fig. 5a, b). Based on the TCGA dataset, we observed a positive correlation of *CPT1A* with *SOX2*, *c-Myc*, *NRF2* or *GPX4* transcripts in NSCLC patients (Fig. 5i). Finally, we developed a better prognostic indicator for NSCLC patients by using the *CPT1A*^high^*c-Myc*^high^*FBXW7*^low^ expression pattern (Supplementary Fig. 5c). Taken together, our results reveal a novel mechanism indicating that the CPT1A/c-Myc loop prevents LCSCs’ ferroptosis by activating the NRF2/GPX4 antioxidative system and restricting the production of PUFA-PLs through ACSL4. We discovered a mutually reinforcing loop between CPT1A and c-Myc, which played a crucial role in maintaining stemness and resistance to ferroptosis in LCSCs.

### CPT1A-mediated ferroptosis resistance in LCSCs relies on TAMs-derived L-carnitine

**Fig. 6:**
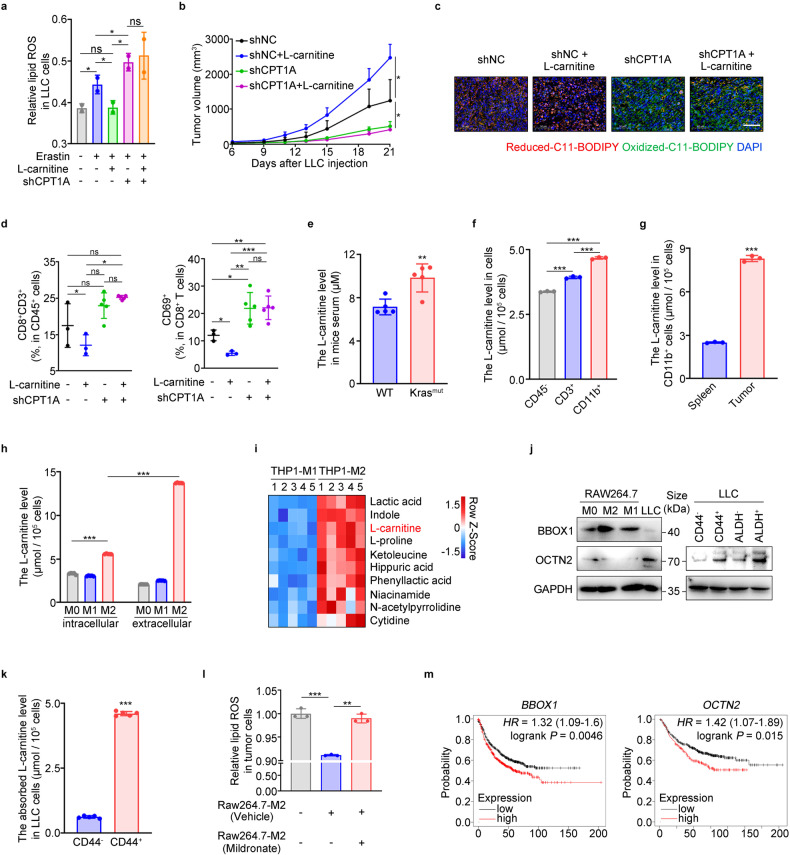
CPT1A-mediated ferroptosis resistance in LCSCs relies on TAMs-derived L-carnitine. **a** Lipid ROS levels in LLC-shNC/shCPT1A cells treated with L-carnitine (10 mM) or not for 2 days and then erastin (2 μM) for 6 h determined by flow cytometry. Data represent the mean ± s.d.; *n* = 2 samples. Effects of L-carnitine (IG, 100 mg/kg) on tumor progression in tumor-bearing mice inoculated with LLC-shNC/shCPT1A cells. Tumor growth curve (data represent the mean ± s.e.m.; *n* = 5 samples) (**b**). Representative images of C11-BODIPY staining in the tumor. Scale bar: 50 μm (insets) (**c**). Percentages of CD8^+^ or CD69^+^CD8^+^ cells in the tumor. Data represent the mean ± s.d.; *n* = 3-5 samples (**d**). **e** L-carnitine levels in the serum of *Kras* mutant or wild type mice. measured by colorimetry. Data represent the mean ± s.d.; *n* = 5 samples.L-carnitine levels in the virous cell types of tumor-bearing mice measured by colorimetry. L-carnitine levels in CD45^-^/CD11b^+^/CD3^+^ cells isolated from the tumor (**f**) and in macrophages isolated from the spleen or the tumor (**g**) in mice. Data represent the mean ± s.d.; *n* = 3 samples. **h** L-carnitine levels of intracellular or extracellular of M0/M1/M2-macrophages measured by colorimetry. Data represent the mean ± s.d.; *n* = 3 samples. **i** Heatmap of metabolites up-regulated in the THP-1-M2 medium calculated by LC-MS (*P* < 0.05; one-way ANOVA) (*n* = 5 samples). **j** Representative western blot for BBOX1 and OCTN2 in RAW264.7-M0/M1/M2 cells or LLC cells (left). Representative western blot for OCTN2 in CD44^+/-^ cells or ALDH^+/-^ cells of LLC cells (right). **k** Absorbed L-carnitine levels in CD44^+/−^ cells treated with L-carnitine or not for 2 days measured by colorimetry. Data represent the mean ± s.d.; *n* = 5 samples. **l** Lipid ROS levels in LLC cells co-cultured with Raw264.7-M2 cells (treated with mildronate or not) or not determined by flow cytometry. Data represent the mean ± s.d.; *n* = 3 samples. **m** Overall survival of NSCLC patients stratified according to tumor BBOX1 or OCTN2 expression analyzed with Kaplan-Meier Plotter websites. Research model of CPT1A/c-Myc loop regulating ferroptosis in LCSCs. *P < 0.05, **P < 0.01, ***P < 0.001; ns: not significant. The statistical analysis was performed using a two-tailed Student’s *t*-test or Pearson’s correlation test

With the assistance of L-carnitine, CPT1A facilitates the transport of long-chain fatty acids into the mitochondria for β-oxidation and energy production. We hypothesized that the L-carnitine/CPT1A axis could suppress ferroptosis in LCSCs. Our results supported this hypothesis as L-carnitine effectively suppressed erastin-induced ferroptosis in LLC-shNC cells but not in LLC-shCPT1A cells (Fig. 6a). Furthermore, we observed that L-carnitine enhanced the stemness phenotype of lung cancer cells, and high doses of L-carnitine (100 mg/kg) significantly promoted tumor progression while suppressing tumoral ferroptosis and CD8^+^ T cell activation in mice (Supplementary Fig. 6a–g). Importantly, the accelerated tumor growth induced by L-carnitine disappeared after CPT1A knockdown, accompanied by restored ferroptosis and reactivated CD8^+^ T cells in the tumor (Fig. 6b–d and Supplementary Fig. 6h), highlighting the strong dependency of L-carnitine on CPT1A as an oncometabolite.

Furthermore, we investigated the source of L-carnitine in tumors and found a marked elevation in L-carnitine concentration in the serum of mice with the *Kras*^G12D^ genotype (Fig. 6e). Subsequent analysis revealed that L-carnitine levels were highest in CD11b^+^ cells compared to CD3^+^ cells or CD45^-^ cells in mouse tumors (Fig. 6f). Moreover, L-carnitine levels were higher in myeloid (CD11b^+^) cells isolated from mouse tumors compared to those from the mouse spleen (Fig. 6g). Notably, L-carnitine levels, particularly extracellular levels, were significantly elevated in macrophages with an M2 phenotype (Fig. 6h and Supplementary Fig. 6i). Metabolomics analysis using Liquid chromatography-mass spectrometry (LC-MS) revealed that L-carnitine was highly abundant in M2 macrophages’ conditioned medium below 3 kDa (Fig. 6i). Further investigation into the synthesis and absorption of L-carnitine revealed that BBOX1 was highly expressed in RAW264.7, BMDMs, THP-1, or TAMs with an M2 phenotype, while OCTN2 was mainly expressed in lung tumor cells, especially in LCSCs, which have a superior capacity to absorb extracellular L-carnitine (Fig. 6j, k and Supplementary Fig. 6j–l). Importantly, we found that erastin-induced ferroptosis was suppressed in lung cancer cells co-cultured with M2 macrophages but significantly restored when M2 macrophages were treated with mildronate, a BBOX1 inhibitor (Fig. 6l). Additionally, lung cancer patients with high expression of BBOX1 or OCTN2 exhibited worse survival rates (Fig. 6m). These findings collectively suggest that L-carnitine, synthesized and secreted from TAMs, is absorbed by LCSCs and, in conjunction with CPT1A, acts as a ferroptosis suppressor in lung cancer.

### Targeting CPT1A improves ICB therapeutic efficiency in lung cancer

**Fig. 7:**
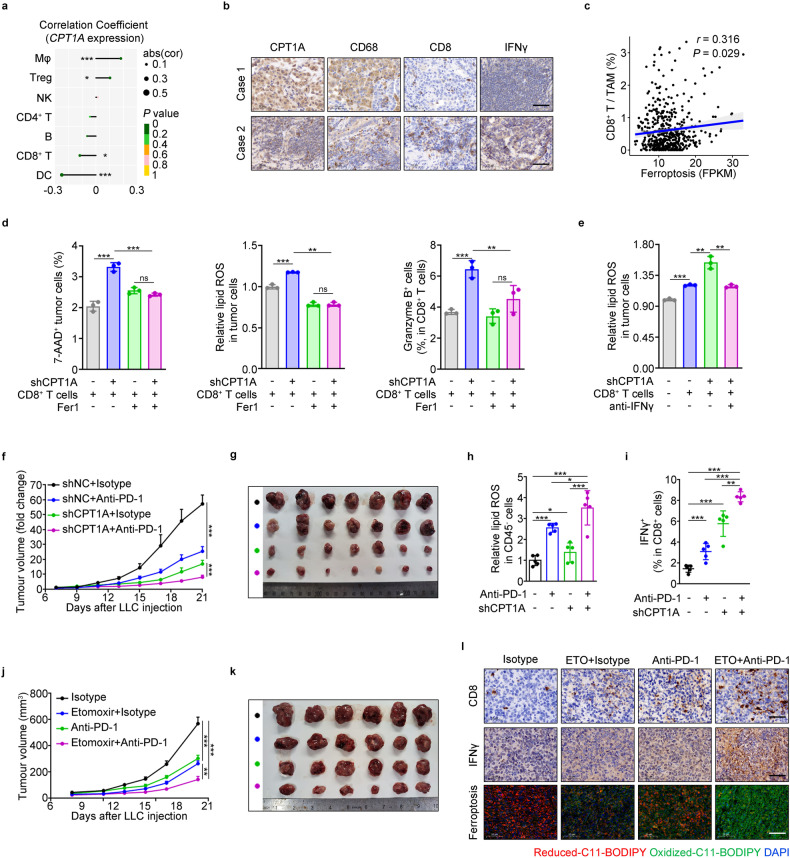
Targeting CPT1A improves ICB therapeutic efficiency in lung cancer. **a** Correlation analysis of immune cells infiltration in tumor with *CPT1A* expression in NSCLC patients. **b** Representative IHC staining for CPT1A, CD68, CD8 and IFNγ in the lung tissues from NSCLC patients. Scale bar: 50 μm (inserts). **c** Correlation analysis of the CD8^+^ T cells/TAMs infiltration with ferroptosis in NSCLC patients by bioinformatic analysis. **d** Percentage of 7-AAD^+^ cells or lipid ROS levels in LLC-shNC/shCPT1A cells and percentage of granzyme B^+^ cells in CD8^+^ T cells (isolated from the spleen of tumor-bearing mice) in the presence of Fer1 or not for 2 days determined by flow cytometry. **e** Lipid ROS levels in LLC-shNC/shCPT1A cells co-cultured with CD8^+^ T cells (isolated from the spleen of tumor-bearing mice) or not while treated with IFNγ neutralizing antibodies or vehicle for 2 days determined by flow cytometry. Effects of PD-1 antibody on tumor progression in tumor-bearing mice inoculated with LLC-shNC/shCPT1A cells (*n* = 7). Tumor growth curve (data represent the mean ± s.e.m.) (**f**). Photograph of the mouse tumor (**g**). Lipid ROS levels of CD45^-^ cells in mouse tumor (**h**). Percentage of IFNγ^+^CD8^+^ T cells in the mouse tumor (**i**). Effects of PD-1 antibody and etomoxir on tumor progression in tumor-bearing mice inoculated with LLC-shNC/shCPT1A cells (*n* = 6). Tumor growth curve (data represent the mean ± s.e.m.) (**j**). Photograph of the mouse tumor (**k**). Representative images of C11-BODIPY staining and IHC staining for CD8 and IFNγ in the mouse tumor. Scale bar: 50 μm (inserts) (**l**). Data represent the mean ± s.d.; scale bar: 50 μm. *P < 0.05, **P < 0.01, ***P < 0.001. The statistical analysis was performed using a two-tailed Student’s *t*-test or Pearson’s correlation test

Our findings indicated that tumor-intrinsic CPT1A plays a critical role in restraining the infiltration and activation of CD8^+^ T cells (Figs. 1g, h, 7a, b and Supplementary Fig. 7a, b). Given the strong correlation between the high ratio of CD8^+^ T cells to TAMs and enhanced tumoral ferroptosis in lung cancer patients (Fig. 7c), and the fact that T cell-induced tumoral ferroptosis is a primary mechanism in tumor regression during immune checkpoint blockade (ICB) therapy, we explored the potential value of targeting CPT1A in lung cancer treatment. Our results demonstrated that CPT1A in tumor cells effectively restrains CD8^+^ T cell-induced tumor ferroptosis, which can be markedly suppressed by Fer1 or IFNγ neutralizing antibody in vitro (Fig. 7d, e). These findings imply that tumor-intrinsic CPT1A acts as a critical gatekeeper, impeding CD8^+^ T cell-induced tumoral ferroptosis in lung cancer.

Subsequently, we implemented a therapeutic approach targeting CPT1A in combination with a PD-1 neutralizing antibody in a mouse model (Supplementary Fig. 7c). The combined treatment significantly inhibited tumor growth and increased tumoral ferroptosis levels compared with treatment alone (Fig. 7f–h and Supplementary Fig. 7d). It also promoted the infiltration and activation of CD8^+^ T cells while decreasing the proportion of M2 macrophages in the tumor or spleen (Fig. 7i and Supplementary Fig. 7e). Furthermore, etomoxir combined with PD-1 antibody had a similar effect on tumor suppression in mice (Fig. 7j–l and Supplementary Fig. 7f, g). Additionally, we found that CPT1A expression was significantly higher in lung cancer patients with progressive disease than in those with a partial response to anti-PD-1 therapy (Supplementary Fig. 7h). These findings suggest a crucial role of CPT1A-induced ferroptosis resistance in LCSCs to evade attack by cytotoxic T cells. Targeting CPT1A may serve as a critical mechanism for improving tumor immunotherapy in lung cancer.

**Fig. 8:**
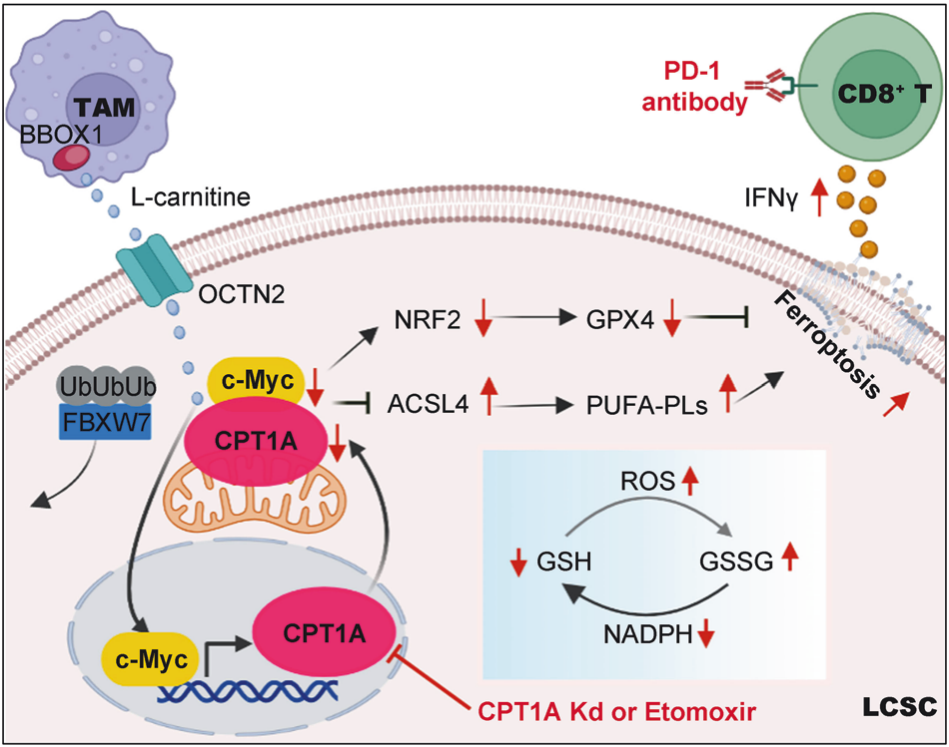
Targeting CPT1A-mediated metabolic remodeling induces ferroptosis and synergizes with immunotherapy to inhibit tumor progression. TAMs-derived L-carnitine activates the CPT1A/c-Myc positive feedback loop, further enhancing the antioxidative capacity through NRF2/GPX4 pathway and restricting the production of PL-PUFAs by downregulating ACSL4 expression to prevent ferroptosis in LCSCs. Targeting CPT1A-mediated metabolic remodeling induces ferroptosis and synergizes with tumor immunotherapy for lung cancer. Schematic was created with BioRender (www.biorender.com)

In conclusion, our study reveals the central role of the CPT1A/c-Myc feedforward circuit activated by TAMs-derived L-carnitine in governing the ferroptosis resistance of LCSCs and promoting immune evasion during cancer progression. We also demonstrate a novel mechanism indicating that the CPT1A/c-Myc loop prevents LCSCs’ ferroptosis by activating the NRF2/GPX4 antioxidative system and restricting the production of PUFA-PLs through ACSL4. Our findings provide a metabolic context in which CPT1A suppression can create therapeutic opportunities by triggering ferroptosis in treating ICB-tolerant cancers (Fig. 8).

## Discussion

During the process of ferroptosis, tumor cells heavily rely on their abnormal metabolism and cellular redox state.^7^ However, the regulatory role of CPT1A, which is the rate-limiting enzyme in FAO metabolism, in the ferroptosis of LCSCs remains unknown. Our current study unveils a positive feedback loop between CPT1A and c-Myc, which activates the NRF2/GPX4 cellular antioxidative system and reduces the production of PUFA-PLs by ACSL4, consequently suppressing ferroptosis in LCSCs. Targeting CPT1A-mediated metabolic remodeling induce ferroptosis and synergize it with immunotherapy to effectively inhibit tumor progression. These findings shed light on the central role of CPT1A-mediated metabolic remodeling in governing the resistance of LCSCs to ferroptosis and facilitating immune evasion during cancer progression.

In recent years, accumulating evidence suggests that combining conventional chemotherapeutic agents or immunotherapy with targeting lipid metabolism is a promising strategy for overcoming drug resistance.^15,32,33^ Studies have indicated a significant upregulation of CPT1A expression in breast cancer or leukemia,^16,34^ where it could promote tumor progression and metastasis by inhibiting apoptosis or anoikis.^31,35^ In contrast, our results revealed that CPT1A-mediated FAO was a crucial metabolic characteristic of LCSCs, and the suppression of CPT1A promoted ferroptosis sensitivity but not apoptosis in LCSCs. This heterogeneous mechanism of CPT1A promoting tumor cell survival suggests that targeting CPT1A to induce ferroptosis in LCSCs may be an effective strategy for NSCLC therapy. We also observed that LCSCs exhibit lower levels of ROS, which is consistent with a previous study in hematologic malignancy.^31^ These ROS^low^ subpopulations were also found to be more resistant to ferroptosis in our study. Although FAO metabolism could regulate the cellular redox state closely linked to lipid peroxidation and ferroptosis,^6^ the exact role of CPT1A in ferroptosis was previously overlooked. Our findings indicate that CPT1A promotes the accumulation of antioxidants, such as NADPH or GSH, and restricts the production of PUFA-PLs to prevent ferroptosis in lung cancer cells. These findings suggest that targeting CPT1A provides a new approach for eliminating ferroptosis-resistant subpopulations in NSCLC.

The transcription factor c-Myc plays a vital role in tumorigenesis, metabolism, CSCs, immune escape, and even ferroptosis in cancer.^36,37^ However, c-Myc proteins lack a druggable conformation for small molecule binding,^36^ which necessitates the exploration of superior strategies for targeting c-Myc-related networks. Our study revealed a novel mechanism whereby the direct interaction of CPT1A with c-Myc impedes FBXW7-mediated ubiquitination degradation of c-Myc, while c-Myc transcriptionally activates CPT1A expression to form a positive feedback regulation. This mechanism is analogous to the Aurora B kinase (AURKB)/c-Myc positive feedforward loop that reinforces c-Myc-regulated oncogenic programs.^38^ A previous study also found that targeting CPT1A with etomoxir impeded the progression of MYC-driven triple-negative breast cancer.^14^ In addition to the accumulation of antioxidants, such as NADPH or GSH, we found that CPT1A could upregulate GPX4 expression and decrease PUFA-PLs levels to prevent ferroptosis in LCSCs. However, the unexpected increase in the expression of ferroptosis suppressor protein 1 (FSP1) upon CPT1A down-regulation was observed (data not shown), and we propose that it might be a self-protective response in lung cancer cells. Our study highlights the mechanism of the CPT1A/c-Myc loop in regulating the expression of GPX4 and ACSL4, supporting the rationality of targeting CPT1A to promote ferroptosis in lung cancer.

Recently, metabolites secreted by TAMs have emerged as a driver of tumor growth and resistance to therapy.^39,40^ However, the role of macrophage-tumor cell metabolic interactions in dictating the therapeutic efficiency of ICB in lung cancer is unknown. L-carnitine, the substrate of CPT1A, is highly abundant in tumors,^31,41,42^ and increased intake of red meat (rich in L-carnitine) is associated with increased cancer mortality.^43^ Our results reveal that L-carnitine, acting with CPT1A to suppress tumor ferroptosis, accelerates tumor progression in mice. Furthermore, L-carnitine in tumors is mainly secreted by TAMs and taken up by LCSCs, suggesting that TAMs derived L-carnitine may function as an oncometabolite and hold potential as a diagnostic biomarker for lung cancer patients. Additionally, it has been shown that the critical proteins in L-carnitine synthesis (BBOX1) and transport (OCTN2) are highly expressed in tumors.^44,45^ We find that BBOX1 and OCTN2 were highly expressed in M2-like macrophages and LCSCs, respectively, implying the need for further studies to explore their essential functions in lung cancer progression. Metabolic adaptation in tumor cells dramatically influences the success of immune-based therapies,^46^ and we find that tumor CPT1A correlates with poor responses in cancer patients treated with checkpoint blockades. As an immunogenic cell death, tumoral ferroptosis can cooperate with cancer immunity in various mouse tumor models.^28^ In our study, targeting CPT1A can cooperate with CD8^+^ T cells to induce tumoral ferroptosis, leading to practical tumor regression. Therefore, CPT1A suppression might be a previously unappreciated approach to synergize ferroptosis in LCSCs with immunotherapy.

This study highlights the crucial role of CPT1A-mediated metabolic reprogramming in sustaining the persistence and immune evasion of LCSCs. However, we acknowledge several limitations of this study. There is still limited understanding of the mechanism on synthesis and secretion of L-carnitine from TAMs and its potential as an indicator of antitumor immune response in clinical settings. Furthermore, the exact molecular domain of c-Myc interacting with CPT1A and other mechanisms of CPT1A regulating the stability of c-Myc protein should be explored in future. Finally, more investigations are necessary to unravel the metabolic network involving L-carnitine/CPT1A in different cellular contexts, including T cells, macrophages, and various tumor types. Therefore, additional studies are warranted to elucidate the influence of metabolic regulation on tumor immunology and enhance the efficacy of cancer immunotherapy.

In conclusion, our study reveals that CPT1A exerts a significant influence on ferroptosis resistance in LCSCs, driving complex metabolic reprogramming. Furthermore, we uncover a novel feedback regulatory mechanism between CPT1A and c-Myc during the process of ferroptosis in LCSCs, shedding light on how metabolic rewiring supports tumor cell survival during immune clearance. These findings offer new insights for the development of a potential therapeutic strategy aimed at disrupting ferroptosis resistance in LCSCs to inhibit tumor progression, with promising clinical implications. Targeting CPT1A represents a previously overlooked approach to synergistically induce ferroptosis in LCSCs in combination with immunotherapy for lung cancer.

## Materials and methods

### Cell culture

We sourced the mouse lewis lung cancer (LLC) cell line and the human embryonic kidney cell line HEK293T from the Cell Bank of the Chinese Academy of Sciences located in Shanghai, China. These cells were cultured at 37 °C with 5% CO_2_ in DMEM medium supplemented with 10% fetal bovine serum (FBS), 100 U/mL penicillin, and 0.1 mg/mL streptomycin. We also obtained the human NSCLC cell lines H1299 and A549, the human monocytic cell line THP-1, and the mouse macrophage RAW264.7 cell line from ATCC and cultured them according to recommended guidelines. We regularly conducted mycoplasma contamination tests on all cell lines used in this study, and we are pleased to report that all results were negative. The cell lines underwent authentication through cellular morphology and short tandem repeat analysis conducted by Microread Inc. in Beijing, China.

Mouse CD8^+^ T cells were isolated from spleens using the EasySep™ Mouse CD8^+^ T Cell Isolation Kit (Stemcell Technologies, Cambridge, MA). These isolated T cells were then stimulated with anti-CD3 (Clone 145-2C11, BD Biosciences, San Jose, CA) and anti-CD28 (Clone 37.51, BD Biosciences) antibodies for a duration of 2 days. Primary bone marrow-derived macrophages (BMDMs) were prepared from mice as previously described. To induce polarization of macrophages into the M1 subset, BMDMs, RAW264.7 cells, or THP-1 cells (pretreated with 200 ng/mL PMA) were exposed to 100 ng/mL LPS and 20 ng/mL IFNγ. For polarization into the M2 subset, BMDMs, RAW264.7 cells, or THP-1 cells (pretreated with 200 ng/mL PMA) were incubated with 20 ng/mL IL4 and 20 ng/mL IL-13. Supplementary Table S1 provides a summary of the key resources used in this process.

### Animals

The *Sftpc*-creER^T2^; *Kras*^LSL-G12D/+^; *Cpt1a*^Flox/Flox^ transgenic mice were used in this study to specifically knockout the *Cpt1a* gene in type II alveolar epithelial cells of the lung. This was achieved through the Cre-FloxP system, where loxP fragments were inserted using the CRISPR system and homology-directed repair. The deletion of exon 9 resulted in a frame-shift mutation, leading to the inactivation of the *Cpt1a* gene. To initiate tumor formation in these mice, Tamoxifen was administered twice a week for 2 months via intraperitoneal injection. Tamoxifen was dissolved in corn oil at a concentration of 20 mg/ml, and each injection contained 75 mg/kg of body weight. This treatment activated the oncogenic *Kras*^G12D^ and facilitated the conditional deletion of *Cpt1a*.

In this study, male C57BL/6J or NOD/SCID mice aged 6-8 weeks were used for the LLC tumor graft model. The mice were randomly assigned to each group with a minimum of 5 mice per group. To form subcutaneous tumors, 1 × 10^6^ LLC cells expressing shCtrl, shCPT1A#1, and shCPT1A#2 separately were injected subcutaneously into each mouse. Tumor volume was calculated using the formula: (width^2^ × length)/2, and tumors were analyzed histologically, immunologically, and for survival at different time points. In order to test the efficacy of anti-PD-1 antibody treatment, BioXcell’s mouse anti-PD-1 antibody or mouse anti-IgG2a antibody was administered via intraperitoneal injections at a dosage of 100 μg/injection on days 7, 10, 13, 16, and 19 after tumor inoculation. When the tumors reached a size of approximately 80 mm^3^, the mice were randomly divided into four groups for erastin treatment. Mice were intraperitoneally injected with 20 mg/kg erastin every day for 15 days. For L-carnitine treatment, once the tumors reached about 80 mm^3^, the mice were orally treated with L-carnitine at a dosage of 100 mg/kg per day (Sigma-Aldrich) or saline (200 μL) for 21 days (*n* = 6-7). Animal studies were conducted in accordance with institutional guidelines and with the authorization of the Experimental Animal Welfare and Ethics Committee of Basic Medical Sciences Institute of Chinese Academy of Medical Sciences (ACUC-A02-2022-114).

### Stable cell strain establishment

To achieve stable knockdown of mouse *Cpt1a* in LLC cells or homo *CPT1A* in H1299/A549 cells, we utilized the pLV2N-U6-Puro vector (GenePharma Co., Ltd., Shanghai, China) to insert control or *CPT1A*-targeting shRNA templates. These shRNA templates were named shNC, shCPT1A#1, and shCPT1A#2. Lentiviruses expressing these shRNAs were used to infect tumor cells, and subsequently, clone selection was performed using puromycin at a concentration of 2 μg/mL. This process allowed us to establish stable cell lines with CPT1A knockdown. Supplementary Table S2 provides the specific sequences of the shRNAs.

### Tumor sphere formation assay

After preparing single cell suspensions, the cells were enumerated and seeded onto 6-well ultra-low attachment culture plates (Corning, USA) at an optimal density. These plates were filled with complete Mammocult medium (Stem Cell Technologies, Canada). Following a 7-day incubation period, the tumorspheres with a diameter of at least 70 μm were quantified.

### ATP production measurement

To determine the level of ATP production, we utilized the cell titer-blue cell viability assay kit (Promega, USA) in accordance with the manufacturer’s instructions. The resulting values were normalized to the protein concentration.

### RNAi/overexpression experiment

*c-Myc* siRNA was transiently transfected using Lipo2000 according to the manufacturer’s instructions. The siRNAs were synthesized by Beijing Qingke Biological Company. pCDNA3.1-3FLAG-MYC plasmid (Tsingke Biotechnology Co., Ltd.) was transiently transfected using Lipo2000 according to the manufacturer’s instructions.

### Quantitative PCR analysis

The Trizol reagent (Invitrogen) was employed for the extraction of total RNA from tissue or cells. The concentration of RNA was determined using a NanoDrop spectrometer. Subsequently, 1000 ng of total RNA was subjected to reverse transcription using the Prime Script® RT reagent Kit Perfect Real Time kit (TaKaRa) to generate cDNA. For RNA-time PCR analysis, SYBR-Green fluorescent dye (Biorad) was utilized, and the reactions were performed on a Biorad CFX 96 instrument. The specific primer sequences used can be found in supplementary Table S2.

### Histology and immunohistochemistry

Tissue chips containing 75 pairs of lung adenocarcinoma and para-cancerous tissues or 80 lung adenocarcinoma tissues were purchased from SHANGHAI OUTDO BIOTEC CO., LTD (LUC1505, LUC1601). Lung adenocarcinoma tissues of two NSCLC patients were obtained from Tianjin Medical University General Hospital. Tumor tissue from patients or mice was fixed in 4% paraformaldehyde. Tissues were embedded with paraffin and sectioned by microtome. The slides were stained with hematoxylin and eosin (H&E) using standard protocol. For CPT1A or c-Myc immunohistochemistry, slides of various tissue were blocked with goat serum for 1 h. Subsequently, the slides were incubated with anti-CPT1A (1:200; 15184-1-AP, Proteintech) anti-c-Myc (1:200; 10828-1-AP, Proteintech), anti-FBXW7 (1:200; 28424-1-AP, Proteintech), anti-GPX4 (1:200; ab125066, Abcam) or anti-ACSL4 (1:200; ab155282, Proteintech) overnight at 4 °C followed by detection with the microscope (Leica). Hematoxylin (ZSGB-BIO) was used as counterstain. The human samples were obtained with informed consent, and the study was approved by Ethics Committee of Basic Medical Sciences Institute of Chinese Academy of Medical Sciences (2019029). The study was conducted in accordance with recognized ethical guidelines. The clinicopathological characteristics of NSCLC patients are listed in Supplementary Tables S3 and S4.

### Immunofluorescence (IF)

Immunofluorescence (IF) was performed following a previous protocol^47^ and using primary antibodies raised against CPT1A (1:50; 15184-1-AP, Proteintech) or c-Myc (1:50; 10828-1-AP, Proteintech).

### Western bolt

The whole cell lysates were prepared following established protocols. In summary, cells were lysed using M-PER Mammalian Protein Extraction Reagent (Thermo Scientific Technologies, USA) supplemented with a protease inhibitor cocktail (Thermo Scientific Technologies, USA). The resulting lysates contained total protein. The protein concentration in the lysates was measured using a Bradford assay kit (Beyotime Biotechnology, Shanghai). Antibodies for CPT1A, c-Myc, SOX2, OCT4, NANOG, GPX4, ACSL4, NRF2, Ubiqutin, FBXW7, GAPDH and β-actin (Cell Signaling Technology; 1:1000), were used for western bolt.

### Flow cytometry

To detect cells with high ALDEFLUOR activity, we conducted the ALDEFLUOR assay (Stemcell Technologies) in accordance with the manufacturer’s instructions. The assay involved incubating cells in the presence or absence of the ALDH inhibitor diethylaminobenzaldehyde for 40 min at 37 °C. Following this, single cells were exposed to CD44 and other primary antibodies in PBS with 1% FBS for 30 min at 4 °C. Finally, cells were stained with 7-AAD, which was used to exclude nonviable cells.

### BODIPY-C11 staining

For BODIPY-C11 staining, cells were suspended in 1 mL of Hanks Balanced Salt Solution (HBSS, Gibco 14-025-092) containing either 3 mM BODIPY^TM^ 581/591 C11 or BODIPY^TM^ 665/676. The suspension was then incubated for 30 min at 37 °C in a tissue culture incubator. After incubation, the cells were washed and resuspended in 200 mL of fresh HBSS. Subsequently, the stained cells were immediately analyzed using a flow cytometer (LSR II, BD Biosciences). In the case of BODIPY^TM^ 581/591 C11 staining, both non-oxidized C11 (PE channel) and oxidized C11 (FITC channel) signals were monitored. The ratio of the mean fluorescence intensity (MFI) of FITC to the MFI of PE was calculated for each sample. To account for variations, the data were normalized to control samples, as indicated by relative lipid ROS levels.

### Chromatin immunoprecipitation (ChIP)

ChIP was carried out using the EZ-Zyme Chromatin Prep Kit (Millipore, Billerica, MA, USA) according to the manufacturer’s protocol. Anti-c-Myc antibody was used to precipitate DNA crosslinked with c-Myc, and anti-mouse IgG was also used as a negative control. qPCR was performed to detect DNA fragments of the *CPT1A* promoter region. The primers used are listed in Supplementary Table S2.

### Luciferase reporter assay

We co-transfected 0.5 mg of pGL4.10 vector expressing *CPT1A*-BS1 or *CPT1A*-BS2 (or the indicated mutant), along with 0.5 mg of pRL-TK plasmid (as a reference) and 50 ng of Renilla luciferase reporter into H1299 cells using Lipofectamine 2000. This transfection was performed in triplicate. Luciferase activity was measured 24 hours later using the Dual-Luciferase Reporter Assay System from Promega. Firefly luciferase activities were normalized to Renilla luciferase control values and presented as an average of the triplicates.

### Seahorse XF24 respirometry

XF24 plates were seeded with 2 × 10^4^ per well of H1299 shNC / shCPT1A sphere cells and allowed to stabilize overnight. The extracellular acidification rate (ECAR) was measured using the XF24 extracellular flux analyzer, utilizing the glucose stress fuel flex test kits from Agilent. The ECAR measurements were carried out following the manufacturer’s instructions. Subsequently, the data obtained were analyzed using Wave software provided by Seahorse/Agilent.

### Transmission electron microscopy (TEM)

The samples were prepared as follows: first, the samples were embedded in 10% gelatin and fixed in glutaraldehyde at 4 °C; subsequently, the fixed samples were sectioned into blocks of less than 1 mm in size. The blocks were then dehydrated using a series of alcohol concentrations (30%, 50%, 70%, 90%, 95%, and 100%) for 10 min each. Following dehydration, the samples were infiltrated with Quetol-812 epoxy resin mixed with propylene oxide at increasing concentrations (25%, 50%, 75%, and 100%) for 3 h each step. Finally, the samples were embedded in pure, fresh Quetol-812 epoxy resin and polymerized at different temperatures (35 °C for 12 h, 45 °C for 12 h, and 60 °C for 24 h). Ultrathin sections (100 nm) were obtained using a Leica UC6 ultramicrotome and stained with uranyl acetate for 10 min and lead citrate for 5 min at room temperature. The samples were observed using an FEI Tecnai T20 TEM transmission electron microscope.

### Quantification of L-carnitine or NADPH

For analysis of intracellular nucleotides, cells (1 × 10^6^) or cell suspension were homogenized in 100 μL of Assay Buffer and centrifuged at 13,000 *g* for 10 min to remove insoluble materials. The resulting sample was brought to 50 μL/well with Assay Buffer in a 96-well plate, and the manufacturer’s instructions (Biovision, K642-100) were then followed. To detect NADP, NADPH, and their ratio, the NADP/NADPH Assay Kit (Abcam, ab65349) was used.

### LC-MS analysis of metabolites

H1299-shNC or H1299-shCPT1A cells were cultured in 100 mm dishes until they reached approximately 60–70% density. Metabolites were then extracted from the cells using cold 80% methanol, followed by two washes with PBS. The collected supernatants were spun at 13,300 rpm for 10 min at 4 °C and subsequently subjected to vacuum freeze-drying. The metabolites were then analyzed using a Dionex Ultimate 3000 UPLC system coupled to a TSQ Quantiva Ultra triple-quadrupole mass spectrometer (Thermo Fisher, CA) equipped with a heated electrospray ionization (HESI) probe. Separation of the extracts was performed using a synergi Hydro-RP column (2.0 × 100 mm, 2.5 mm, Phenomenex) with a binary solvent system consisting of 10 mM tributylamine adjusted with 15 mM acetic acid in water (mobile phase A) and methanol (mobile phase B). A 25-min gradient from 5% to 90% mobile phase B was used for the analysis. Metabolites were detected using selected reaction monitoring (SRM) in positive-negative ion switching mode with a resolution of 0.7 FWHM for both Q1 and Q3. The source voltage was set to 3500 v for positive ion mode and 2500 v for negative ion mode. Source parameters included a capillary temperature of 350 °C, heater temperature of 300 °C, sheath gas flow rate of 35, and auxiliary gas flow rate of 10. Metabolite identification and peak integration were performed using Tracefinder 3.2 (Thermo, USA).

### Bioinformatics analysis

RNA sequencing data from NSCLC patients who received anti-PD-1/PD-L1 treatment were obtained from the GEO accession GSE135222 and GSE126044. The CIBERSORTx online analysis platform (https://cibersortx.stanford.edu/) provided a means to evaluate the relative proportions of immune cells infiltrating the tumors. To assess mRNA expression, the GDC TCGA Lung Adenocarcinoma (LUAD) database (HiSeqV2) was accessed through the UCSC Xena platform (https://xena.ucsc.edu). Z-scores were then calculated based on all tumor samples for further analysis. Different gene signatures were determined by averaging the z-scores of specific genes. For example, the apoptosis gene signature was derived from the average z-scores of *BIM, PUMA, BID, BMF, BAD, HRK, BIK*, and *NOXA*. Necroptosis gene signature was determined by averaging the z-scores of *RIPK1, RIPK3*, and *MLKL*. Autophagy gene signature was derived from the average z-scores of *MAP1LC3B, BECN1*, and *SQSTM1*, while the ferroptosis gene signature was determined by averaging the z-scores of *ALOX5, LPCAT3, ACSL4, TFRC*, and *SLC11A2*. Pearson’s correlation analysis was performed using RStudio. Overall survival (OS) data along with the expression levels of *CPT1A, c-Myc*, and *FBXW7* genes were obtained from the UCSC Xena platform (https://xena.ucsc.edu). Survival curves were generated using the R packages “survival” and “survminer”.

### Statistical analysis

The data were analyzed using GraphPad Prism 8.0. Statistical significance was determined by performing an unpaired two-tailed Student’s *t*-test to compare two groups or by conducting one-way or two-way ANOVA with Tukey’s correction for multiple comparisons when comparing three or more groups. A *P* value less than 0.05 was considered statistically significant. The survival of mice in the experiments was represented using Kaplan-Meier curves and statistical significance was estimated using a log-rank test.

### Supplementary information

Revised Supplementary Information

original and uncropped films of Western blots

## Data Availability

The corresponding authors made the data utilized in the present investigation accessible to interested individuals upon a reasonable request. The transcriptome data has been submitted to the GSA-HUMAN database (HRA006509).

## References

[1] Siegel RL, Miller KD, Fuchs HE, Jemal A (2022). Cancer statistics, 2022. CA Cancer J. Clin..

[2] Clara JA, Monge C, Yang Y, Takebe N (2020). Targeting signalling pathways and the immune microenvironment of cancer stem cells-a clinical update. Nat. Rev. Clin. Oncol..

[3] Tang R (2020). Ferroptosis, necroptosis, and pyroptosis in anticancer immunity. J. Hematol. Oncol..

[4] Miao Y (2019). Adaptive immune resistance emerges from tumor-initiating stem cells. Cell.

[5] Baldominos P (2022). Quiescent cancer cells resist T cell attack by forming an immunosuppressive niche. Cell.

[6] Zheng J, Conrad M (2020). The metabolic underpinnings of ferroptosis. Cell Metab..

[7] Lei G, Zhuang L, Gan B (2022). Targeting ferroptosis as a vulnerability in cancer. Nat. Rev. Cancer.

[8] Wu M (2022). Cancer stem cell-regulated phenotypic plasticity protects metastasized cancer cells from ferroptosis. Nat. Commun..

[9] Liu CC (2021). Esophageal cancer stem-like cells resist ferroptosis-induced cell death by active Hsp27-GPX4 pathway. Biomolecules.

[10] Wu X (2022). Co-loaded lapatinib/PAB by ferritin nanoparticles eliminated ECM-detached cluster cells via modulating EGFR in triple-negative breast cancer. Cell Death Dis..

[11] Zhou X (2023). Molecular mechanisms of ROS-modulated cancer chemoresistance and therapeutic strategies. Biomed. Pharmacother..

[12] Fan F (2021). A Dual PI3K/HDAC inhibitor induces immunogenic ferroptosis to potentiate cancer immune checkpoint therapy. Cancer Res..

[13] Yang F (2023). Ferroptosis heterogeneity in triple-negative breast cancer reveals an innovative immunotherapy combination strategy. Cell Metab..

[14] Camarda R (2016). Inhibition of fatty acid oxidation as a therapy for MYC-overexpressing triple-negative breast cancer. Nat. Med..

[15] Oren Y (2021). Cycling cancer persister cells arise from lineages with distinct programs. Nature.

[16] Tang M (2022). CPT1A-mediated fatty acid oxidation promotes cell proliferation via nucleoside metabolism in nasopharyngeal carcinoma. Cell Death Dis..

[17] Jiang N (2022). Fatty acid oxidation fuels glioblastoma radioresistance with CD47-mediated immune evasion. Nat. Commun..

[18] Liu Z (2023). CPT1A-mediated fatty acid oxidation confers cancer cell resistance to immune-mediated cytolytic killing. Proc. Natl. Acad. Sci. USA.

[19] Ahmed N, Escalona R, Leung D, Chan E, Kannourakis G (2018). Tumour microenvironment and metabolic plasticity in cancer and cancer stem cells: perspectives on metabolic and immune regulatory signatures in chemoresistant ovarian cancer stem cells. Semin. Cancer Biol..

[20] Zhu P (2022). 5-hydroxytryptamine produced by enteric serotonergic neurons initiates colorectal cancer stem cell self-renewal and tumorigenesis. Neuron.

[21] Xue Y (2023). Intermittent dietary methionine deprivation facilitates tumoral ferroptosis and synergizes with checkpoint blockade. Nat. Commun..

[22] Liao P (2022). CD8(+) T cells and fatty acids orchestrate tumor ferroptosis and immunity via ACSL4. Cancer Cell.

[23] Console L (2020). Carnitine traffic in cells. Front. Cell Dev. Biol..

[24] Wishart DS (2009). HMDB: a knowledgebase for the human metabolome. Nucleic Acids Res..

[25] Chen C (2015). IKKβ enforces a LIN28B/TCF7L2 positive feedback loop that promotes cancer cell stemness and metastasis. Cancer Res..

[26] Zhou Z (2022). Targeting the macrophage-ferroptosis crosstalk: a novel insight into tumor immunotherapy. Front. Biosci. (Landmark Ed.).

[27] Mihaylova MM (2018). Fasting activates fatty acid oxidation to enhance intestinal stem cell function during homeostasis and aging. Cell Stem Cell.

[28] Wang W (2019). CD8(+) T cells regulate tumour ferroptosis during cancer immunotherapy. Nature.

[29] Wang T (2018). JAK/STAT3-regulated fatty acid β-oxidation is critical for breast cancer stem cell self-renewal and chemoresistance. Cell Metab..

[30] Newton K, Strasser A, Kayagaki N, Dixit VM (2024). Cell death. Cell.

[31] Li C (2022). A metabolic reprogramming amino acid polymer as an immunosurveillance activator and leukemia targeting drug carrier for T-cell acute lymphoblastic leukemia. Adv. Sci..

[32] Yang R, Yi M, Xiang B (2022). Novel insights on lipid metabolism alterations in drug resistance in cancer. Front. Cell Dev. Biol..

[33] Luis G (2021). Tumor resistance to ferroptosis driven by Stearoyl-CoA Desaturase-1 (SCD1) in cancer cells and fatty acid biding protein-4 (FABP4) in tumor microenvironment promote tumor recurrence. Redox Biol..

[34] Gatza ML, Silva GO, Parker JS, Fan C, Perou CM (2014). An integrated genomics approach identifies drivers of proliferation in luminal-subtype human breast cancer. Nat. Genet..

[35] Wang YN (2018). CPT1A-mediated fatty acid oxidation promotes colorectal cancer cell metastasis by inhibiting anoikis. Oncogene.

[36] Dhanasekaran R (2022). The MYC oncogene-the grand orchestrator of cancer growth and immune evasion. Nat. Rev. Clin. Oncol..

[37] Chen C (2019). Targeting LIN28B reprograms tumor glucose metabolism and acidic microenvironment to suppress cancer stemness and metastasis. Oncogene.

[38] Jiang J (2020). Direct phosphorylation and stabilization of MYC by aurora B kinase promote T-cell leukemogenesis. Cancer Cell.

[39] El-Kenawi A (2021). Macrophage-derived cholesterol contributes to therapeutic resistance in prostate cancer. Cancer Res..

[40] Halbrook CJ (2019). Macrophage-released pyrimidines inhibit gemcitabine therapy in pancreatic cancer. Cell Metab..

[41] Ni Y, Xie G, Jia W (2014). Metabonomics of human colorectal cancer: new approaches for early diagnosis and biomarker discovery. J. Proteome Res..

[42] Wang J (2022). CRIP1 suppresses BBOX1-mediated carnitine metabolism to promote stemness in hepatocellular carcinoma. EMBO J..

[43] Zheng Y (2019). Association of changes in red meat consumption with total and cause-specific mortality among US women and men: two prospective cohort studies. BMJ.

[44] Liao C (2020). Identification of BBOX1 as a therapeutic target in triple-negative breast cancer. Cancer Discov..

[45] Juraszek B, Czarnecka-Herok J, Nałęcz KA (2021). Glioma cells survival depends both on fatty acid oxidation and on functional carnitine transport by SLC22A5. J. Neurochem..

[46] Liu Y (2021). Tumors exploit FTO-mediated regulation of glycolytic metabolism to evade immune surveillance. Cell Metab..

[47] Du R (2019). TGIF2 promotes the progression of lung adenocarcinoma by bridging EGFR/RAS/ERK signaling to cancer cell stemness. Signal Transduct. Target. Ther..

