# Supplementary material for: Targeting carnitine palmitoyl transferase 1A (CPT1A) induces ferroptosis and synergizes with immunotherapy in lung cancer
Source: Signal Transduct Target Ther. 2024 Mar 7;9:64. doi: 10.1038/s41392-024-01772-w (PMC10920667; doi:10.1038/s41392-024-01772-w)
Supplement: Supplementary file 1 — Revised Supplementary Information [file 41392_2024_1772_MOESM1_ESM.pdf]

Supplementary Materials for

Targeting carnitine palmitoyl transferase 1A (CPT1A) induces ferroptosis and synergizes with immunotherapy in lung cancer

Lei Ma, Chong Chen, Chunxing Zhao, Tong Li, Lingyu Ma, Jiayu Jiang, Zhaojun Duan, Qin Si, Tsung-Hsien Chuang, Rong Xiang, Yunping Luo

Correspondence to: chenchong86@ibms.pumc.edu.cn; ypluo@ibms.pumc.edu.cn

This PDF file includes:

Figures. S1 to S8
Tables S1 to S4

Supplementary Figure 1

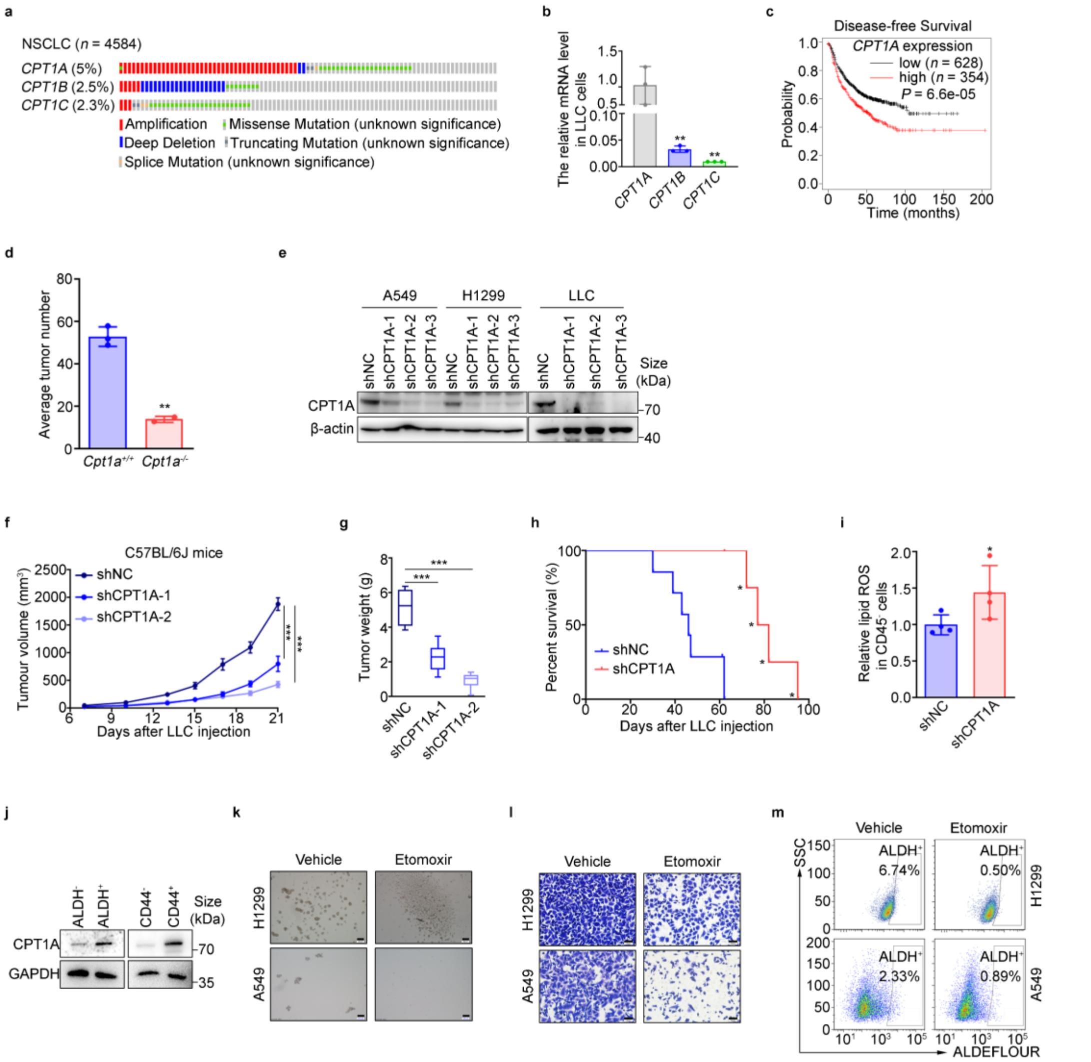

Figure. S1.

CPT1A is essential for LCSCs maintenance and NCSLC progression.

a, Mutation frequency of *CPT1A*, *CPT1B* and *CPT1C* genes in NSCLC patients analyzed with cibioportal website. **b**, Quantification of mRNA for *Cpt1a*, *Cpt1b* and *Cpt1c* in LLC cells. **c**, Disease-free survival of NSCLC patients stratified according to tumor CPT1A expression analyzed with Kaplan-Meier Plotter websites. **d**, Qualification of lung tumor number from SKC (*Sftpc*-CreER^{T2}; *Kras*^{G12D}; *Cpt1a*$^{flox/flox}$) or control mice. **e**, Representative western blot for CPT1A in A549, H1299 and LLC cells. **f-i**, Effects of CPT1A on tumor progression in C57BL/6J mice subcutaneously transplanted with LLC-shNC/shCPT1A cells. Data represent the mean ± s.d., n = 5 samples. Tumor growth curve (**f**). tumor weights (**g**). Overall survival of tumor-bearing mice (**h**). Lipid ROS levels in CD45$^-$ cells in mouse tumor (**i**). **j**, Representative western blot for CPT1A in ALDH$^{+/-}$ or CD44$^{+/-}$ cells of H1299 cells. **k-m**, Effects of etomoxir on stemness phenotypes of H1299 and A549 cells. Sphere-forming assay Scale bars, 200 μm (insets) (**k**). Transwell assay Scale bars, 50 μm (insets) (**l**). Percentage of ALDH$^+$ cells (**m**). *$P < 0.05$, **$P < 0.01$, ***$P < 0.001$; significance was determined

by unpaired two-tailed Student's t test, or one way ANOVA test followed by Tukey's correction.

Supplementary Figure 2

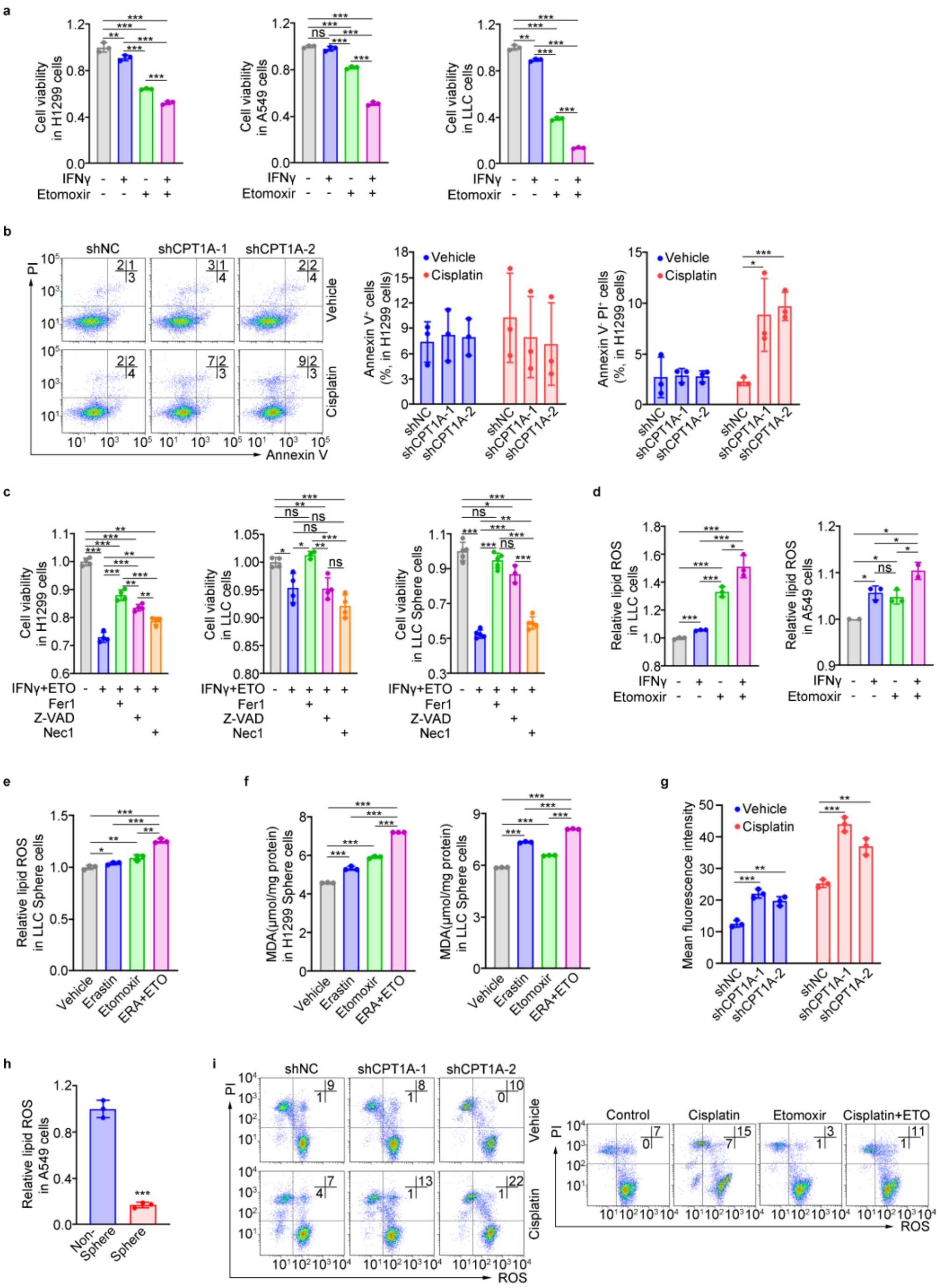

Figure. S2.

CPT1A is an essential driver for the ferroptosis resistance in LCSCs.

a, Cell viability of H1299 (left), A549 (middle) and LLC (right) cells treated with IFNγ (100 μM) or etomoxir (100 μM) for 2 days. **b**, Percentages of AnnexinV$^+$ or AnnexinV$^-$ PI$^+$ cells in H1299-shNC/shCPT1A cells treated with DMSO or cisplain for 2 days. **c**, Cell viability of H1299 cells (left), LLC cells (middle) or LLC sphere cells (right) treated with IFNγ (100 μM) or etomoxir (100 μM) in the presence of ferrostatin-1 (Fer1, 2 mM), necostatin-1 (Nec1, 1 mM), or z-VAD-FMK (z-VAD, 10 mM) for 2 days (n = 3). **d**, Lipid ROS levels in LLC (left) and A549 (right) cells treated with IFNγ (100 μM)

or etomoxir (100 μM) for 2 days determined by flow cytometry. **e**, Lipid ROS levels in LLC sphere cells treated with etomoxir (100 μM) for 2 days and then erastin (2 μM) for 6 h determined by flow cytometry. Data represent the mean ± s.d.; $n = 3$ samples. **f**, The MDA levels in H1299 (left) or LLC (right) sphere cells treated with etomoxir (100 μM) for 2 days and then erastin (2 μM) for 6 h determined by flow cytometry. Data represent the mean ± s.d.; $n = 3$ samples. **g**, Quantification of ROS levels in H1299-shNC/shCPT1A cells treated with DMSO or cisplain for 2 days determined by flow cytometry. Data represent the mean ± s.d.; $n = 3$ samples. **h**, Lipid ROS levels in spheres or non-spheres of A549 cells determined by flow cytometry. **i**, Representative flow cytometry for $ROS^{+}PI^{+}$ or $ROS^{-}PI^{-}$ cells in H1299-shNC/shCPT1A cells treated with DMSO or cisplain for 2 days (left) or H1299 cells treated with etomoxir, cisplatin, or both for 2 days (right). Data represent the mean ± s.d., n = 3-5 samples. *$P < 0.05$, **$P < 0.01$, ***$P < 0.001$; significance was determined by unpaired two-tailed Student's t test, or one way ANOVA test followed by Tukey's correction.

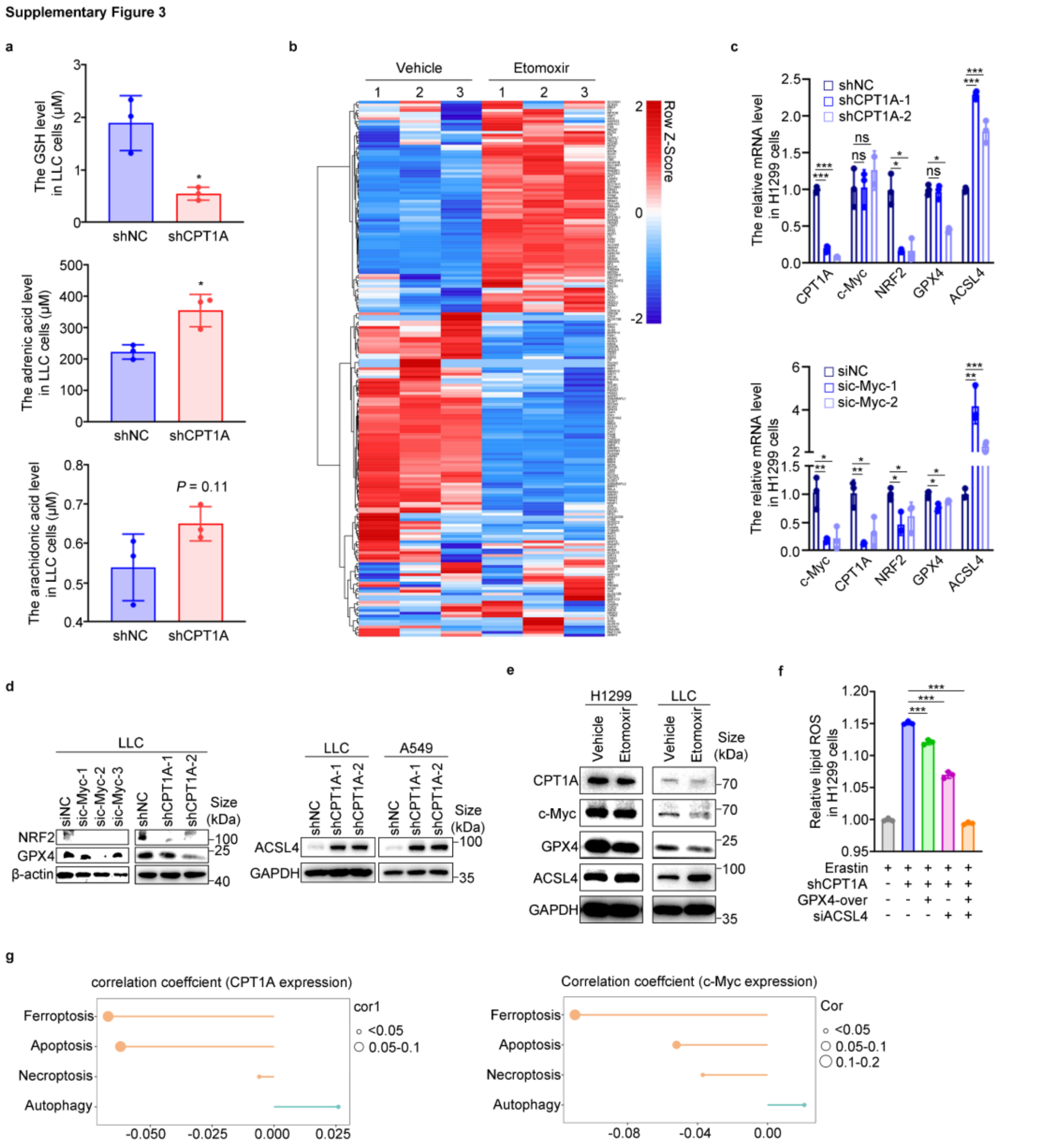

Figure. S3.

CPT1A regulates ferroptosis of LCSCs via GPX4 and ACSL4.

a, Levels of GSH (up), adrenic acid (middle) and arachidomic acid (down) in LLC-shNC/shCPT1A cells calculated by LC-MS. Data represent the mean ± s.d.; $n = 3$ samples. **b**, A heatmap of RNA-seq data depicting the differential genes regulating ferroptosis upon H1299 cells treated with etomoxir (100 μM) or not for 2 days (Q < 0.05). **c**, Quantification of mRNA for *CPT1A*, *c-Myc*, *NRF2, GPX4* and *ACSL4* in H1299-shNC/shCPT1A cells (up) or H1299 cells transfected with sic-Myc or control siRNA (down). **d**, Representative western blot for NRF2 and GPX4 in LLC-shNC/shCPT1A cells or in LLC cells transfected with sic-Myc or control siRNA (left). Representative western blot for ACSL4 in LLC-shNC/shCPT1A cells or A549-shNC/shCPT1A cells (right). **e**, Representative western blot for CPT1A, c-Myc, GPX4 and ACSL4 in H1299/LLC cells treated with etomoxir (100 μM) or not for 2 days. **f**, Lipid ROS levels in H1299-shNC/shCPT1A cells transfected with GPX4 plasmid or

ACSL4 siRNA determined by flow cytometry. Data represent the mean ± s.d., $n = 3$ samples. **g**, Correlation analysis of *CPT1A*/*c-Myc* transcripts with distinct types of cellular death in NSCLC patients by informatic analysis. $*P < 0.05$, $**P < 0.01$, $***P < 0.001$; ns: not significant. The statistical analysis was performed using a two-tailed Student's t-test.

Supplementary Figure 4

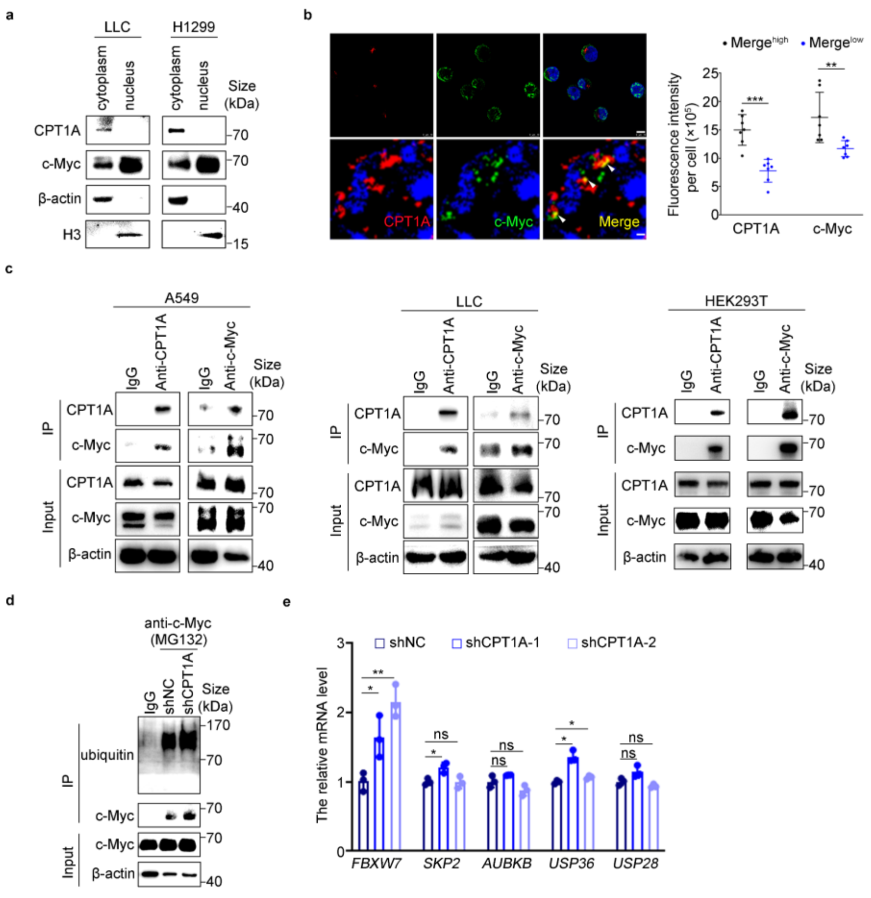

Figure. S4.

CPT1A inhibits ubiquitination degradation of c-Myc.

a, Representative western blot for CPT1A, c-Myc, β-actin and Histone H3 in cytoplasm or nucleus of H1299/LLC cells. **b**, Representative immunofluorescence staining for CPT1A and c-Myc in H1299 cells. Scale bar: 10 μm (up); 0.5 μm (down). Quantifications of immunofluorescence are shown on the right. Data represent the mean ± s.d., n = 7 samples. **c**, Co-immunoprecipitation with CPT1A or c-Myc antibody and representative western blot for CPT1A and c-Myc in A549 (left), LLC (middle) and HEK293T (right) cells. **d**, Co-Immunoprecipitation with c-Myc antibody and representative western blot for ubiquitin and c-Myc in H1299-shNC/shCPT1A cells treated with MG132 (10 μM) for 12 h. **e**, Quantification of mRNA for *FBXW7*, *SKP2*, *AUBKB*, *USP36*, and *USP28* in H1299-shNC/shCPT1A cells. *$P < 0.05$, **$P < 0.01$, ***$P < 0.001$; significance was determined by unpaired two-tailed Student's t test, or one way ANOVA test followed by Tukey's correction.

Supplementary Figure 5

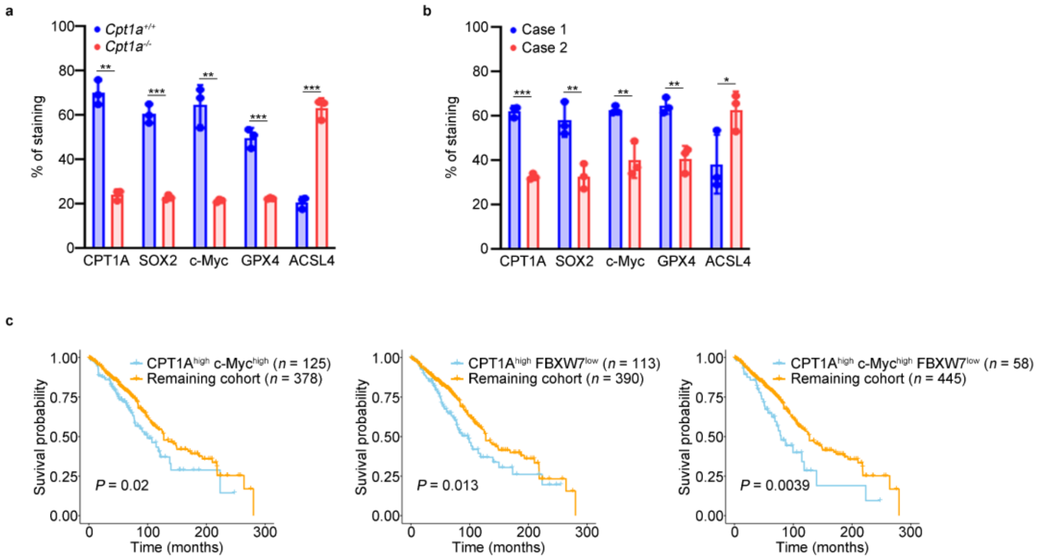

Figure. S5.

The clinical significance of CPT1A, c-Myc and FBXW7 in NSCLC patients.

a, Qualification of IHC staining for CPT1A, SOX2, c-Myc, GPX4 and ACSL4 in the tumor of *Cpt1a*$^{+/+}$ or *Cpt1a*$^{-/-}$ transgenic mice **b**, Qualification of IHC staining for CPT1A, SOX2, c-Myc, GPX4 and ACSL4 in the tumor of NSCLC patients. **c**, Overal survival (OS) of NSCLC patients stratified according to the expression of *CPT1A*, *c-Myc* and *FBXW7*. *P < 0.05, **P < 0.01, ***P < 0.001; ns: not significant. The statistical analysis was performed using a two-tailed Student's t-test, Pearson's correlation test or log-rank (Mantel–Cox) test.

Supplementary Figure 6

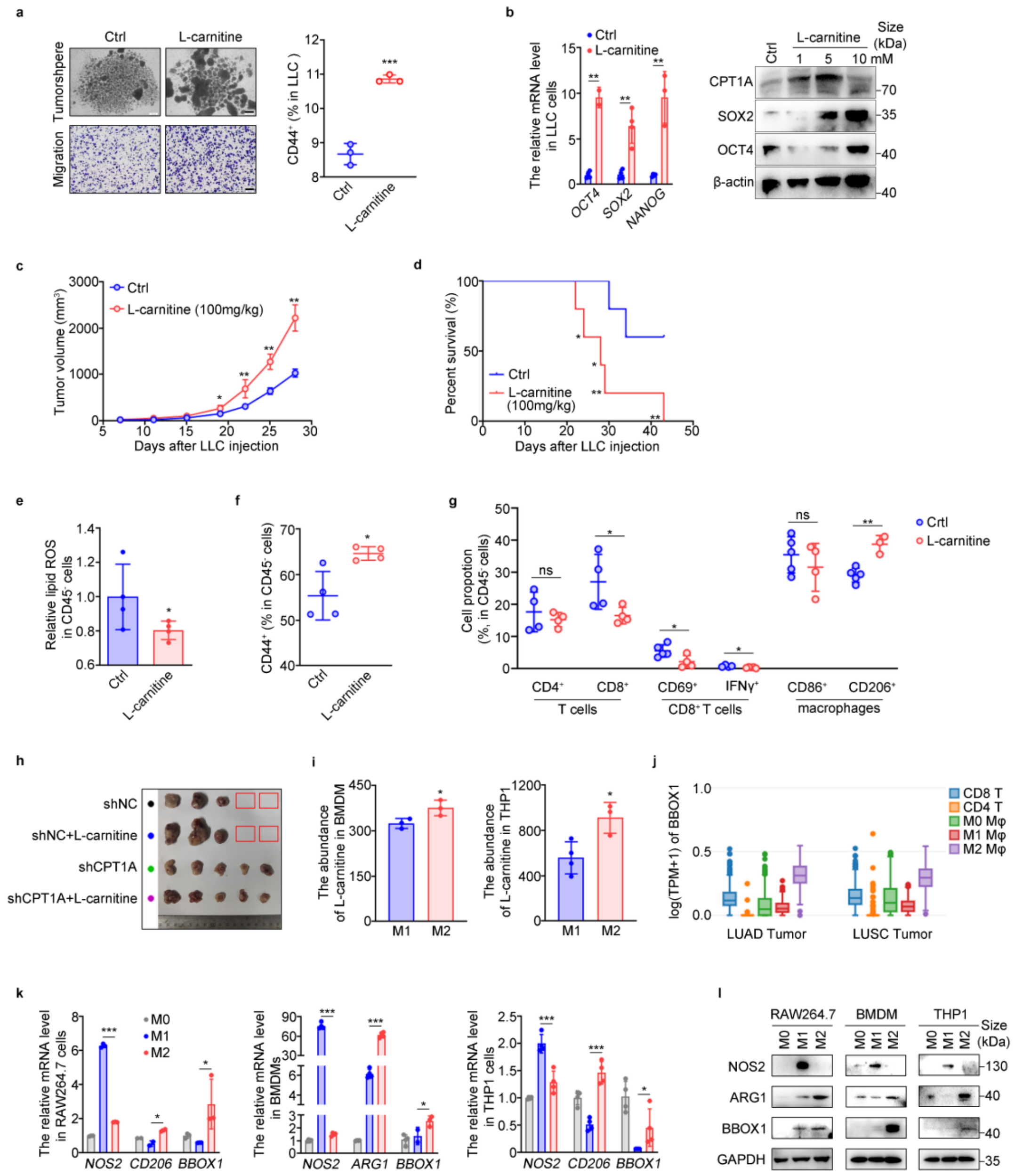

Figure. S6.

L-carnitine promotes cancer stemness and ferroptosis resistance in lung cancer.
a, Effects of L-carnitine on stemness phenotypes of LLC cells. Sphere-forming assay (up). Scale bar: 100 μm. Transwell assay (down). Scale bar: 100 μm. Percentage of $CD44^{+}$ cells (right). **b**, Quantification of mRNA for *OCT4*, *SOX2* and *NANOG* (left) and representative western blot for CPT1A, SOX2 and OCT4 in LLC cells treated with L-carnitine or not. **c-g**, Effects of L-carnitine on tumor progression in tumor-bearing mice inoculated with LLC-shNC/shCPT1A cells (n = 5). C57BL/6J mice subcutaneously transplanted with LLC-shNC/shCPT1A cells ($1{\times}10^{6}$ cells/100 μL), intragastric administration of L-carnitine for 22 days, and sacrificed at day 28. Tumor growth curve (data represent the mean ± s.e.m.) (**c**). Overall survival of tumor-bearing mice (**d**). Lipid ROS levels of $CD45^{-}$ cells in mouse tumor (**e**). Percentage of $CD44^{+}$

tumor cells in the mouse tumor (**f**). Percentage of distinct immune cells in the mouse tumor (**g**). **h**, Tumor photograph of the mice inoculated with LLC-shNC/shCPT1A cells and treated with L-carnitine (IG, 100 mg/kg). **i**, L-carnitine levels in BMDM or THP1 cells calculated by LC-MS. Data represent the mean ± s.d., $n = 3$ samples. **j**, Expression levels of *BBOX1* in $CD8^+$ T cells, $CD4^+$ T cells or macrophages-M0/M1/M2 in the tumor of NSCLC patients, respectively analyzed with TIMER2 website. **k**, Quantification of mRNA for *NOS2*, *CD206*, *ARG1* and *BBOX1* in RAW264.7, BMDM and THP-1 cells. Data represent the mean ± s.d., $n = 3$ samples. **l**, Representative western blot for NOS2, ARG1 and BBOX1 in RAW264.7, BMDM and THP-1 cells. *$P < 0.05$, **$P < 0.01$, ***$P < 0.001$; significance was determined by unpaired two-tailed Student's t test or one way ANOVA test.

Supplementary Figure 7

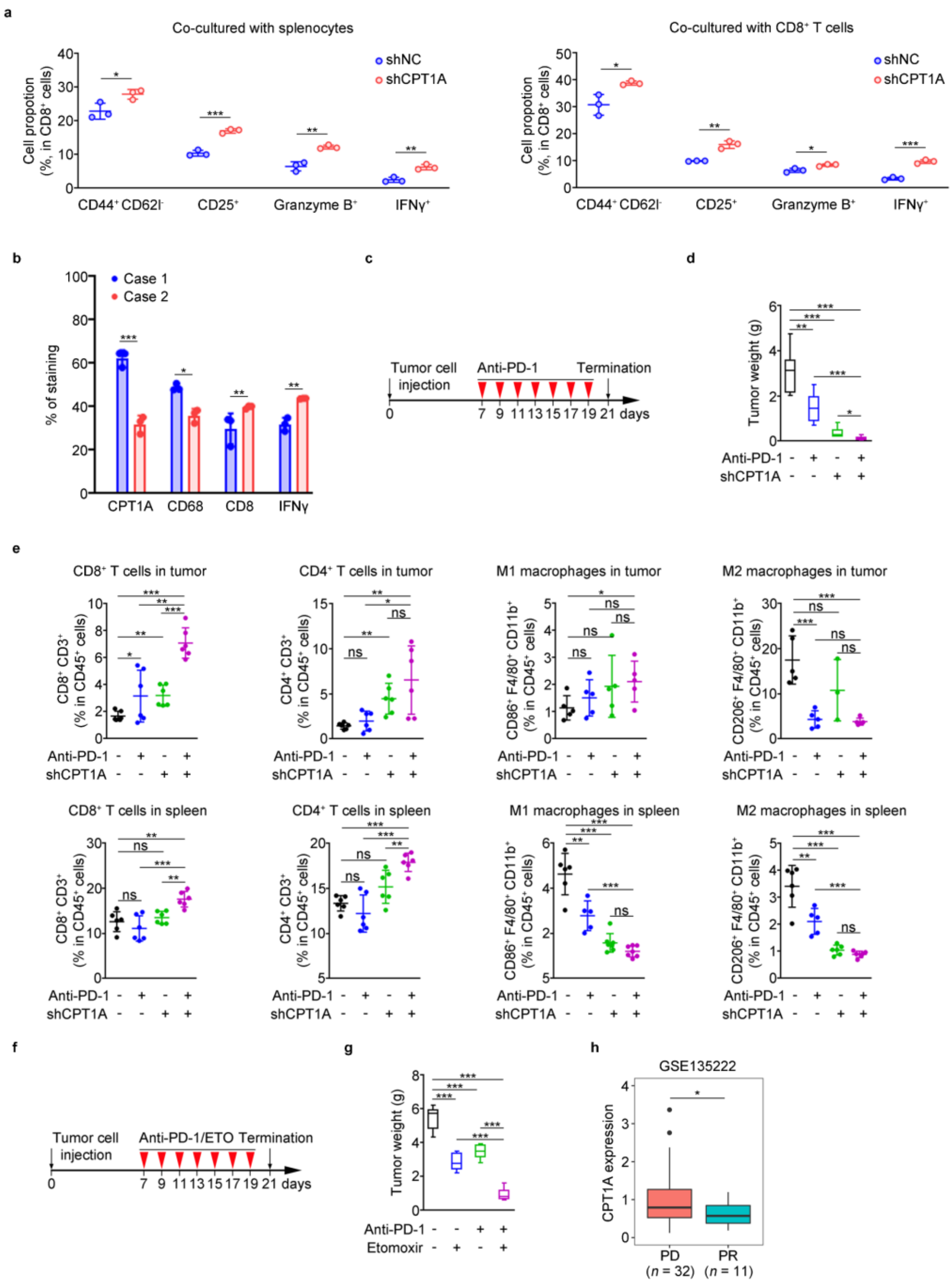

Figure. S7.

Targeting CPT1A promotes the anti-tumoral immunity in lung cancer.

a, Phenotype of CD8$^+$ T cells (isolated from the spleen of tumor-bearing mice) co-cultured with LLC-shNC/shCPT1A cells determined by flow cytometry. **b**, Qualification of IHC staining for CPT1A, CD68, CD8 and IFNγ in the tumor of NSCLC patients. **c-d**, Effects of PD-1 antibody on tumor progression in tumor-bearing mice inoculated with LLC-shNC/shCPT1A cells (*n* = 7). Experimental scheme. C57BL/6J mice were subcutaneously transplanted with LLC-shNC/shCPT1A cells (1×10^6 cells/100 μL), intraperitoneal injection of PD-1 antibody for seven times and were sacrificed at day 21 (**c**). Tumor weight (**d**). **e**, Quantification of CD4$^+$ T cells, CD8$^+$ T

cells, $CD86^+$ macrophages, and $CD206^+$ macrophages gated on $CD45^+$ cells in the tumor or spleen from tumor-bearing mice determined by flow cytometry. **f-g**, Effects of PD-1 antibody and etomoxir on tumor progression in tumor-bearing mice inoculated with LLC-shNC/shCPT1A cells (n = 6). Experimental scheme. C57BL/6J mice were subcutaneously transplanted with LLC-shNC/shCPT1A cells (1×10^6 cells/100 μL), intraperitoneal injection of PD-1 antibody or etomoxir for seven times and were sacrificed at day 21 (**f**). Tumor weight (**g**). **h**, *CPT1A* expression in NSCLC patients with PD-1-blocked immunotherapy by bioinformatic analysis. (PD: progressive disease. PR: partial response). *$P < 0.05$, **$P < 0.01$, ***$P < 0.001$; ns: not significant. The statistical analysis was performed using a two-tailed Student's t-test.

Supplementary Figure 8

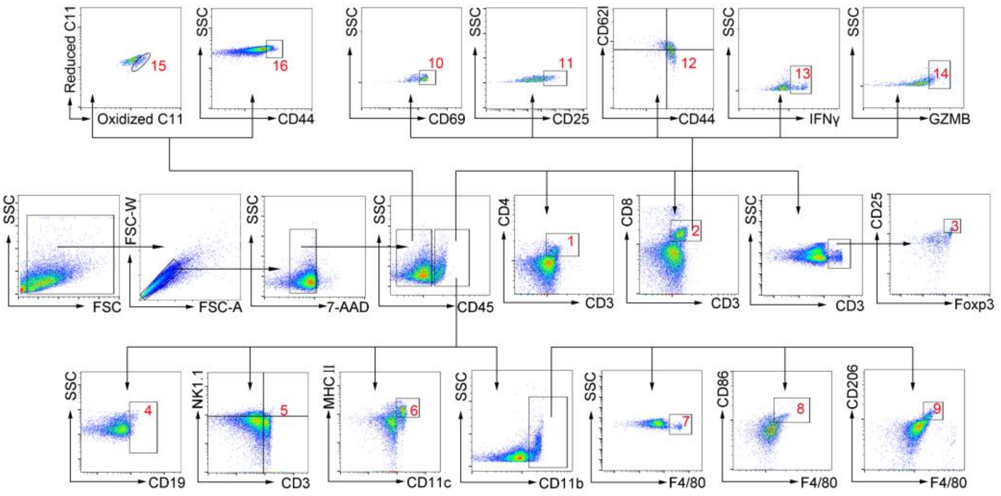

1. CD4$^+$ T 2. CD8$^+$ T 3. Treg 4. B 5. NK 6. DC 7. Macrophage 8. M1 macrophage 9. M2 macrophage
10. CD69$^+$ CD8$^+$ T 11. CD25$^+$ CD8$^+$ T 12. CD44$^+$ CD62l$^-$ T 13. IFNγ$^+$ CD8$^+$ T 14. GZMB$^+$ CD8$^+$ T
15. CD45$^-$ cells of lipid peroxidation 16. CD44$^+$ CD45$^-$ cells

Figure. S8.

Gating strategy for flow cytometry.

Gating strategy for flow cytometry to identify distinct cell populations in mouse tumor or spleen. CD4$^+$ T cells (CD3$^+$, CD8$^+$), CD8$^+$ T cells (CD3$^+$, CD8$^+$), Treg cells (CD3$^+$, CD25$^+$, Foxp3$^+$), B cells (CD19$^+$), NK cells (CD3$^+$, NK1.1$^+$), DC cells (CD11c$^+$, MHC II$^+$), Macrophages (CD11b$^+$, F4/80$^+$), M1 macrophages (CD11b$^+$, F4/80$^+$, CD86$^+$), M2 macrophages (CD11b$^+$, F4/80$^+$, CD206$^+$), CD69$^+$ CD8$^+$ T cells (CD3$^+$, CD8$^+$, CD69$^+$), CD25$^+$ CD8$^+$ T cells (CD3$^+$, CD8$^+$, CD25$^+$), CD44$^+$ CD62L$^-$ CD8$^+$ T cells (CD3$^+$, CD8$^+$, CD44$^+$, CD62L$^-$), IFNγ$^+$ CD8$^+$ T cells (CD3$^+$, CD8$^+$, IFNγ$^+$), GZMB$^+$ CD8$^+$ T cells (CD3$^+$, CD8$^+$, GZMB$^+$) gated on CD45$^+$ cells, lipid ROS level (oxidized C11, reduced C11) and CD44$^+$ cells gated on CD45$^-$ cells.

Table S1.

Key resources tables.

REAGENT or RESOURCE	SOURCE	IDENTIFIER
CPT1A Polyclonal antibody	Proteintech	Cat No: 15184-1-AP
CPT1A Monoclonal antibody	Proteintech	Cat No: 66039-1-Ig
c-MYC Polyclonal antibody	Proteintech	Cat No: 10828-1-AP
c-MYC Monoclonal antibody	Proteintech	Cat No: 67447-1-Ig
SOX2	Abcam	ab59776
OCT4	Abcam	ab19857
NANOG	Cell Signaling Technology	#4903
β-actin Recombinant antibody	Proteintech	Cat No: 81115-1-RR
Anti-FACL4 antibody	Abcam	Cat# ab155282
GPX4	Abcam	ab125066
BBOX1	Abcam	ab189827
OCTN2	Abcam	ab180757
NOX2	Abcam	ab129068
ARG1	Cell Signaling Technology	#93668
GAPDH	Cell Signaling Technology	#5174
Ubiqutin	Proteintech	Cat No: 10201-2-AP
FBXW7	Proteintech	Cat No: 28424-1-AP
NRF2	Proteintech	Cat No: 16396-1-AP
Histone H3	Abcam	ab1791
Normal Rabbit IgG antibody	Cell Signaling Technology	# 2729
Mouse IgG	Proteintech	Cat No: B900620
Anti-Rabbit IgG, Light Chain Specific	Proteintech	Cat No: SA00001-7L
**Chemicals, peptides, and inhibitors**		
L-carnitine	Sigma-Aldrich	C0283
Cytidine	Sigma-Aldrich	C4654
Phenyllactic acid	Sigma-Aldrich	P7251
Hippuric acid	Sigma-Aldrich	112003
Niacinamide	Sigma-Aldrich	N5535
Indole	Sigma-Aldrich	I3408
Sodium palmitate	Sigma-Aldrich	90951
Etomoxir	Sigma-Aldrich	236020
Erastin	MedChemExpress	HY-15763
Ferrostatin-1	MedChemExpress	HY-100579
Necrostatin-1	MedChemExpress	HY-15760

Z-VAD-FMK	MedChemExpress	HY-16658B
Cisplatin	MedChemExpress	HY-17394
BODIPY™ 581/591 C11	Thermo Fisher	Cat# D3861
7-AAD (7-Aminoactinomycin D)	Thermo Fisher	Cat# A1310
PI (Propidium Iodide)	Thermo Fisher	Cat# P1304MP
**Critical commercial assays**		
L-Carnitine Assay kit	Abcam	ab83392
FFA Assay kit	Abcam	ab65341
NADP/NADPH Assay kit	Abcam	ab65349
SimpleChIP® Enzymatic Chromatin IP Kit (Magnetic Beads)	Cell Signaling Technology	Cat# 9003
Lillie's Ferrous Iron Stain Kit	Solarbio	G3320
Nuclear and Cytoplasmic Protein Extraction Kit	Beyotime Biotechnology	P0027
Lipid Peroxidation MDA Assay Kit	Beyotime Biotechnology	S0131S
**Experimental models: Cell lines**		
Mouse cell line: LLC	ATCC	Cat# CRL-1642
Mouse cell line: RAW264.7	ATCC	Cat# TIB-71
Human cell line: THP1	ATCC	Cat# TIB-202
Human cell line: H1299	ATCC	Cat# CRL-5803
Human cell line: A549	ATCC	Cat# CRM-CCL-185
**Experimental models: Organisms/strains**		
Mouse: C57BL/6J	Vital River	NA
Mouse: NOD.SCID	Laboratory	NA
Mouse: transgenic mice	Vital River Laboratory Shanghai Model Organisms Center, Inc.	NA
**Oligonucleotides**		
ChIP-qPCR primers	This paper	listed in Table S1
Mouse-qPCR primers	This paper	listed in Table S1
Human -qPCR primers	This paper	listed in Table S1
**Recombinant DNA**		
pcDNA3.1-CPT1A -His-C	Yixiang Biotechnology	NA

	(Tianjin) Co., Ltd.	
pcDNA3.1-3FLAG-MYC	Tsingke Biotechnology Co., Ltd.	NA
pLV2N-U6-Puro vector	GenePharma Co., Ltd.	listed in Table S1
**Software and algorithms**		
Graphpad Prism 8.0 software	GraphPad Software, Inc	https://www.graphpad.com/
ImageJ	ImageJ: Image Processing and Analysis in Java	https://imagej.nih.gov/ij/
		https://www.flowjo.com/
FlowJo	Tree Star	http://timer.cistrome.org/
Timer 2.0	(Li et al., 2020)	https://doi.org/10.1093/nar-/gkab418
GEPIA	(Li et al., 2021)	
**Deposited Data**		
Gene expression profile of patient	(Jung et al., 2019)	GSE135222
samples after immunotherapy	(Cho et al., 2020)	GSE126044
Transcriptome data	This paper	HRA006509

Table S2.
List of primers used in this study.

Primers for real-time PCR (human)	Primer sequence (5'-3')
CPT1A	F: ATCAATCGGACTCTGGAAACGG<br>R: TCAGGGAGTAGCGCATGGT
SOX2	F: AAAACAGCCCGGACCGCGTC<br>R: CTCGTCGATGAACGGCCGCT
OCT4	F: AAGCGATCAAGCAGCC<br>R: GGAAAGGGACCGAGGAGTA
NANOG	F: ACCTCAGCCTCCAGCAGATGCA<br>R: GGTGCTGAGGCCTTCTGCGT
c-Myc	F: GGCTCCTGGCAAAAGGTCA<br>R: CTGCGTAGTTGTGCTGATGT
ACSL4	F: CATCCCTGGAGCAGATACTCT<br>R: TCACTTAGGATTTCCCTGGTCC
BBOX1	F: CATGGCCCGATGAGCATTACA<br>R: TGAGCCCCAGTATTGGCATTC
SLC16A9	F: CAGGATCACGTTAGCCAATGG<br>R: AAAGAGCCACAGTTTCACCAC
ARG1	F: TGGACAGACTAGGAATTGGCA<br>R: CCAGTCCGTCAACATCAAAACT
MRC1	F: GGGTTGCTATCACTCTCTATGC<br>R: TTTCTTGTCTGTTGCCGTAGTT
GPX4	F: GAGGCAAGACCGAAGTAAACTAC<br>R: CCGAACTGGTTACACGGGAA
SLC7A11	F: TCTCCAAAGGAGGTTACCTGC<br>R: AGACTCCCCTCAGTAAAGTGAC
ACSL3	F: GCCGAGTGGATGATAGCTGC<br>R: ATGGCTGGACCTCCTAGAGTG
LPCAT3	F: GGCTGGATACTATTACACTGCC<br>R: GATCTTTCCCTCCGTCAAAGTAG
NRF2	F: TCAGCGACGGAAAGAGTATGA<br>R: CCACTGGTTTCTGACTGGATGT
FBXW7	F: CGACGCCGAATTACATCTGTC<br>R: CGTTGAAACTGGGGTTCTATCA
USP36	F: CACCACCTCTAGCCAACTACC<br>R: GGCGATCTTTTTCAGGTCTCG
USP28	F: CACTGTTGCTACAGAACCATCT<br>R: TGGGAGACTCCAGTAGACTCA
SKP2	F: ATGCCCCAATCTTGTCCATCT<br>R: CACCGACTGAGTGATAGGTGT
AURKB	F: CGCAGAGAGATCGAAATCCAG<br>R: AGATCCTCCTCCGGTCATAAAA

ALOX15	F: GACCCTGCTATACCAGAGCC<br>R: ACCAGTCCCACTTGTCATCAG
β-actin	F: TCATGAAGTGTGACGTGGACATC<br>R: CAGGAGGAGCAATGATCTTGATCT
GAPDH	F: GGAGCGAGATCCCTCCAAAAT<br>R: GGCTGTTGTCATACTTCTCATGG
**Primers for real-time PCR (mouse)**	
CPT1A	F: TGGTGGTGGGTGTGATAT<br>R: GGTGTCTAGGGTCCGATT
CPT1B	F: GAGACGGACACAGATAGCCC<br>R: TCTTCACTGAGTTCCGATGGG
CPT1C	F: TTCATCTGCGATCCTGACGAC<br>R: CACTGAGGGGTCAATGCACTC
c-Myc	F: GATCCGAACACGGAATATCAGG<br>R: GTCCCCATGCAAGTTAGGAGT
SLC22A5	F: GATCCGAACACGGAATATCAGG<br>R: GTCCCCATGCAAGTTAGGAGT
SOX2	F: GCGGCAACCAGAAAAACAG<br>R: CTCCGTCTCCGACAAAAGT
OCT4	F: GGGCTCTCCCATGCATTCAAAC<br>R: CACCTTCCCTCCAACCAGTTGC
NANOG	F: CACCTTCCCTCCAACCAGTTGC<br>R: GGTGCTGAGGCCTTCTGCGT
INOS	F: CGGAGCCTTTAGACCTCAACA<br>R: CCCTCGAAGGTGAGCTGAAC
ARG1	F: TGTCCCTAATGACAGCTCCTT<br>R: GCATCCACCCAAATGACACAT
BBOX1	F: CTGACAAACGTGGTGAGATCA<br>R: CCACATTGTTGGCATCAATCTTG
GPX4	F: GCCTGGATAAGTACAGGGGTT<br>R: CATGCAGATCGACTAGCTGAG
ACSL4	F: TGAACGTATCCCTGGACTAGG<br>R: TCAGACAGTGTAAGGGGTGAA
LPCAT3	F: CTACCCGTTGGCTCTGTTTTAC<br>R: TGAAGCACGACACATAGCAAG
NRF2	F: CTGAACTCCTGGACGGGACTA<br>R: CGGTGGGTCTCCGTAAATGG
FBXW7	F: TACAAACTGGAGACGAGGAGAA<br>R: CCACAAAACTGTAGGCATGTGAT
SKP2	F: CTACCCGTTGGCTCTGTTTTAC<br>R: TGAAGCACGACACATAGCAAG
β-actin	F: TCATGAAGTGTGACGTGGACATC<br>R: CAGGAGGAGCAATGATCTTGATCT

**Primers for ChIP assay**	
ChIP-hCPT1A-BS1	F: CAGCTTCCGTAGTGCAAACC<br>R: AGATTTCACCGCCCCAAGAG
ChIP-hCPT1A-BS2	F: TGGCCTGTGTCACCAAAATG<br>R: TCCTGGAGCTCCTGATTATCC
**shRNA sequences (human)**	
shNC	TTCTCCGAACGTGTCACGT
shCPT1A-619	GGATGGGTATGGTCAAGATCT
shCPT1A-941	GGATCTGCTGTATATCCTTCC
**shRNA sequences (mouse)**	
ShNC	TTCTCCGAACGTGTCACGT
shCPT1A-2063	GCCTCTATGTGGTGTCCAAGT
shCPT1A-332	GCATGATTGCAAAGATCAATC

Table S3.

Clinicopathological characteristics of patients from NSCLC tumor tissue array.

**Histologic type**	**Characteristics**	**Patients (*n*=155)**
**Pathology staging**	1	21
	2	44
	3	35
	4	11
**Tumor size**	T1	36
	T2	103
	T3	10
	T4	6
**Lymph node metastasis**	N0	100
	N1	38
	N2	17
**Metastasis**	M0	146
	M1	9
**Gender**	Male	71
	Female	84

Table S4.

Clinicopathological characteristics of patients from NSCLC tumor tissue immunohistochemistry.

**Type**	**Case1**	**Case2**
**Histologic type**	Invasive adenocarcinoma	Invasive adenocarcinoma
**Gender**	Female	Male
**Age**	47	65
**Clinical staging**	Ia2	IIa
**Tumor size**	T1b	T2b
**Lymph node metastasis**	N0	N0
**Metastasis**	M0	M0
