# Supplementary material for: Targeting carnitine palmitoyl transferase 1A (CPT1A) induces ferroptosis and synergizes with immunotherapy in lung cancer
Source: Signal Transduct Target Ther. 2024 Mar 7;9:64. doi: 10.1038/s41392-024-01772-w (PMC10920667; doi:10.1038/s41392-024-01772-w)

The raw data of Western blots

Fig.1m

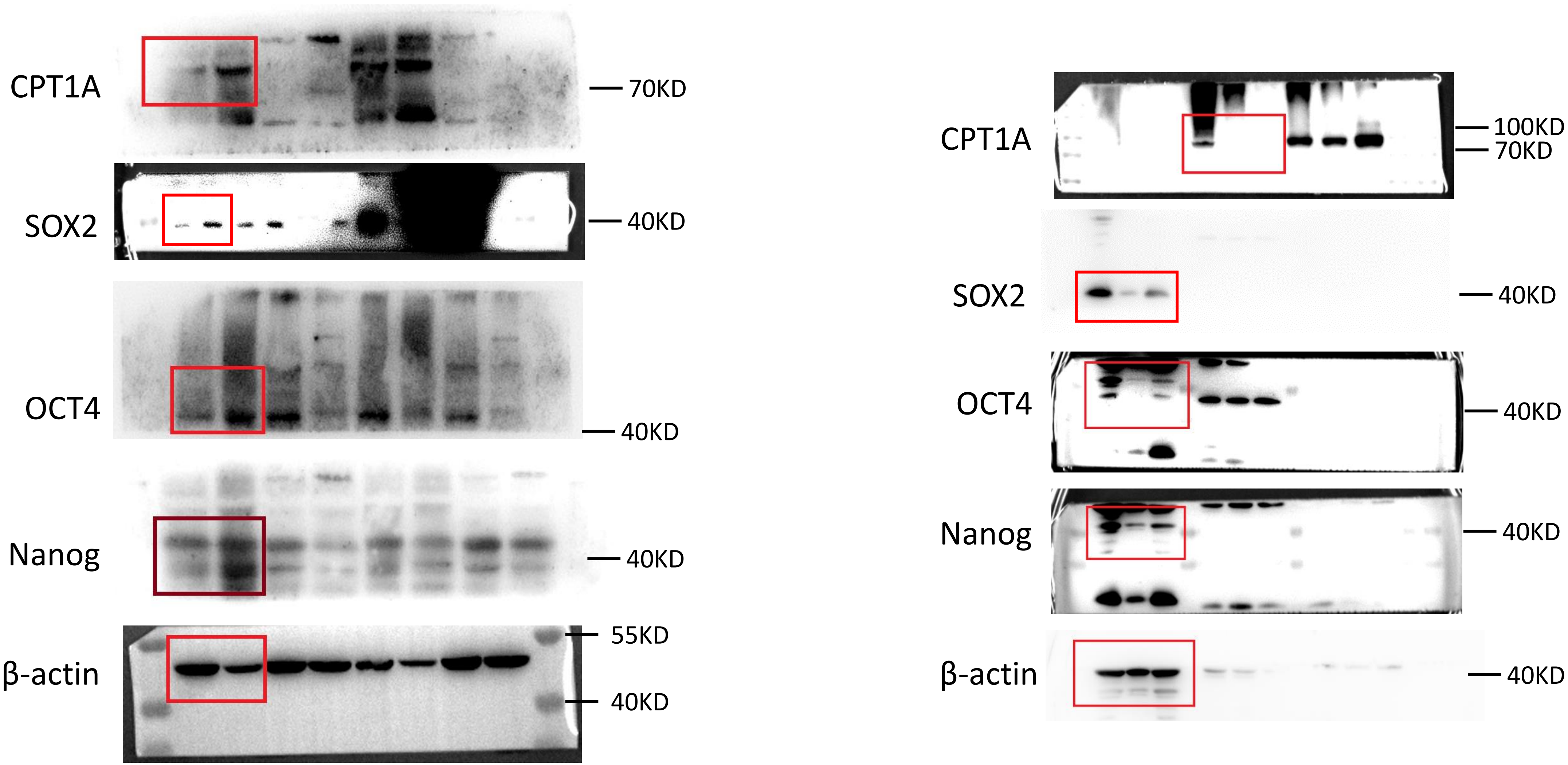

CPT1A
70KD
SOX2
40KD
OCT4
40KD
Nanog
40KD
β-actin
55KD
40KD
CPT1A
100KD
70KD
SOX2
40KD
OCT4
40KD
Nanog
40KD
β-actin
40KD

Fig.4c

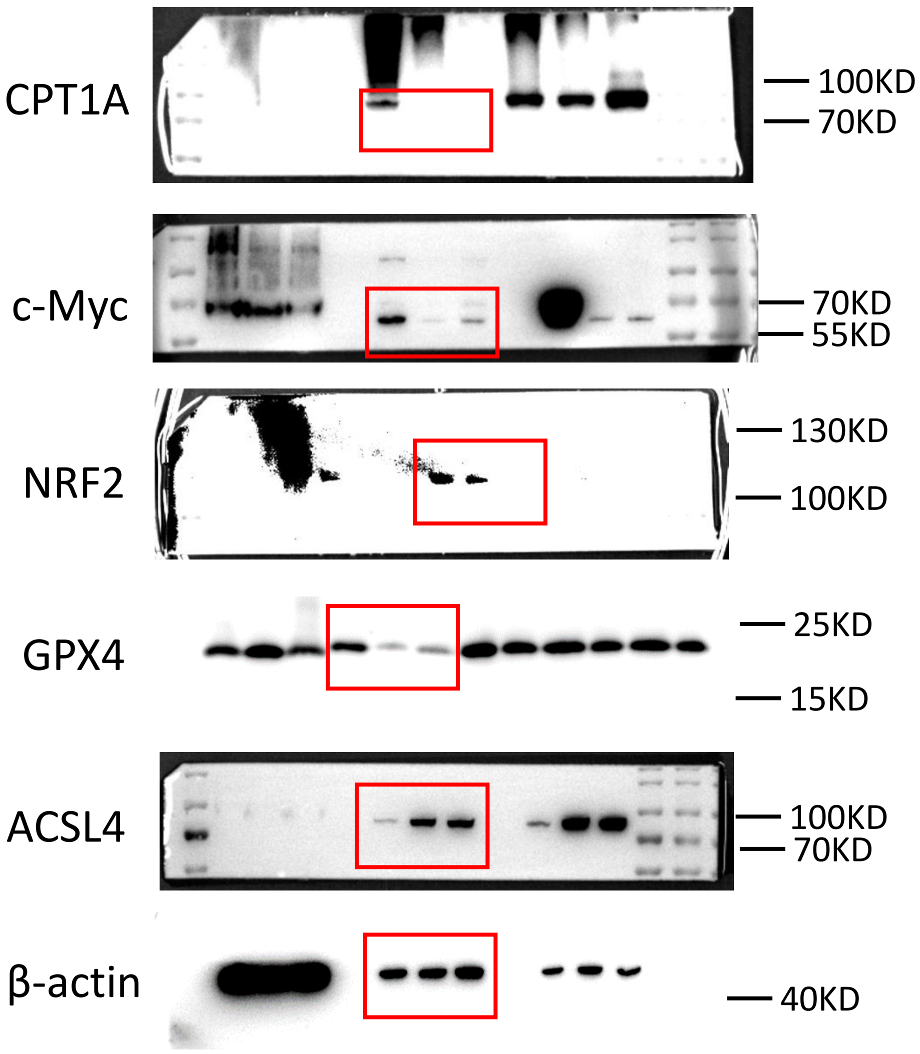
CPT1A
100KD
70KD
c-Myc
70KD
55KD
NRF2
130KD
100KD
GPX4
25KD
15KD
ACSL4
100KD
70KD
β-actin
40KD

Fig.4d

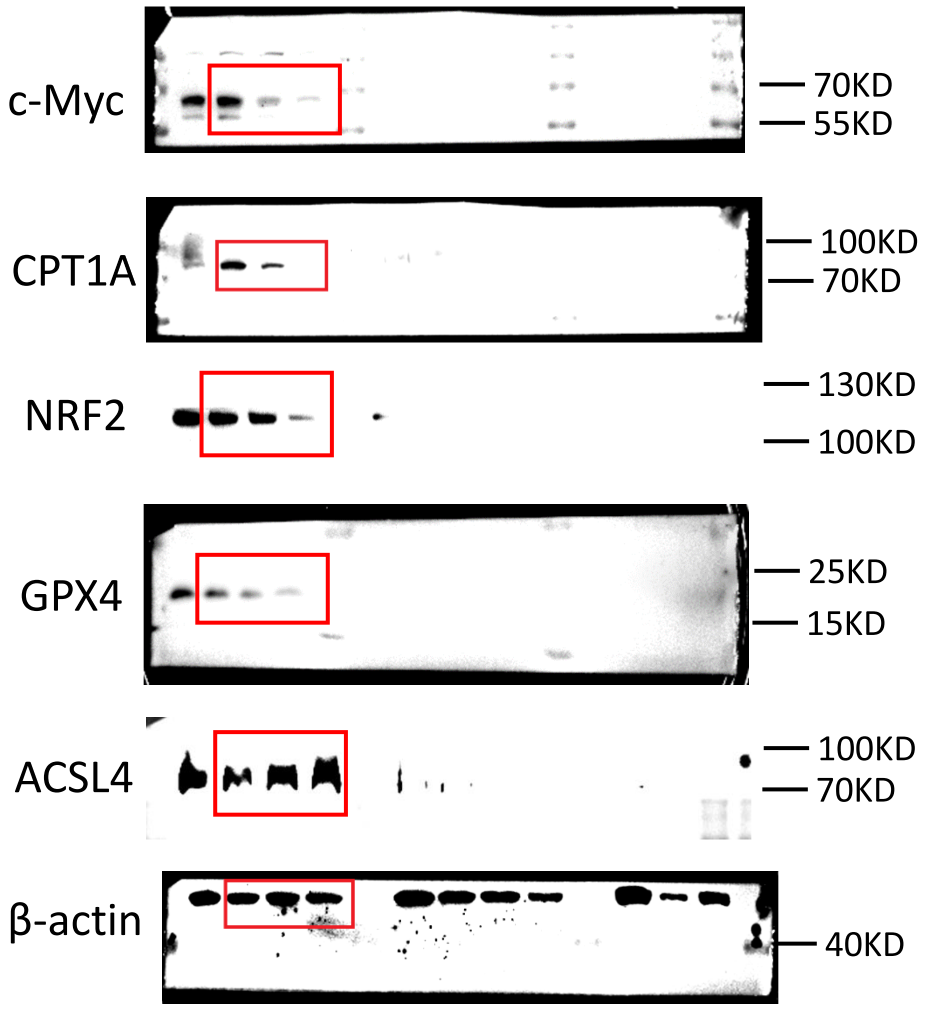
c-Myc
70KD
55KD
CPT1A
100KD
70KD
NRF2
130KD
100KD
GPX4
25KD
15KD
ACSL4
100KD
70KD
β-actin
40KD

Fig.4e

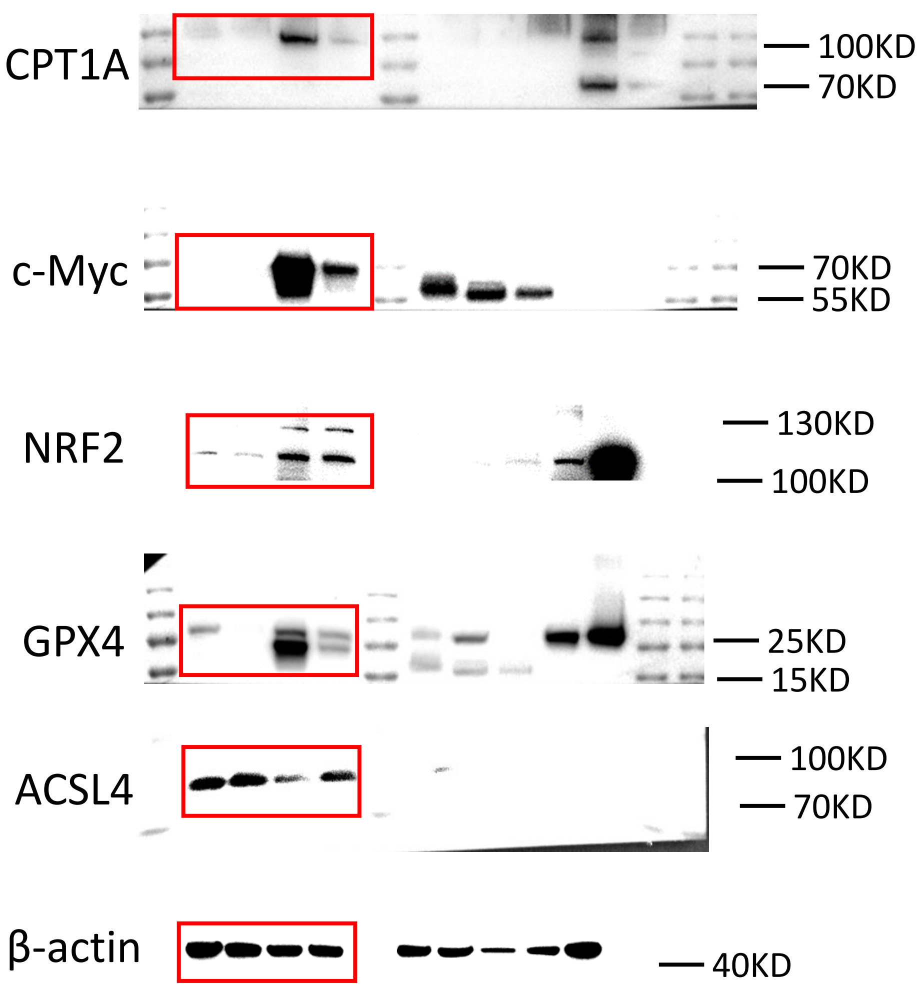
CPT1A
100KD
70KD
c-Myc
70KD
55KD
NRF2
130KD
100KD
GPX4
25KD
15KD
ACSL4
100KD
70KD
β-actin
40KD

Fig.4h

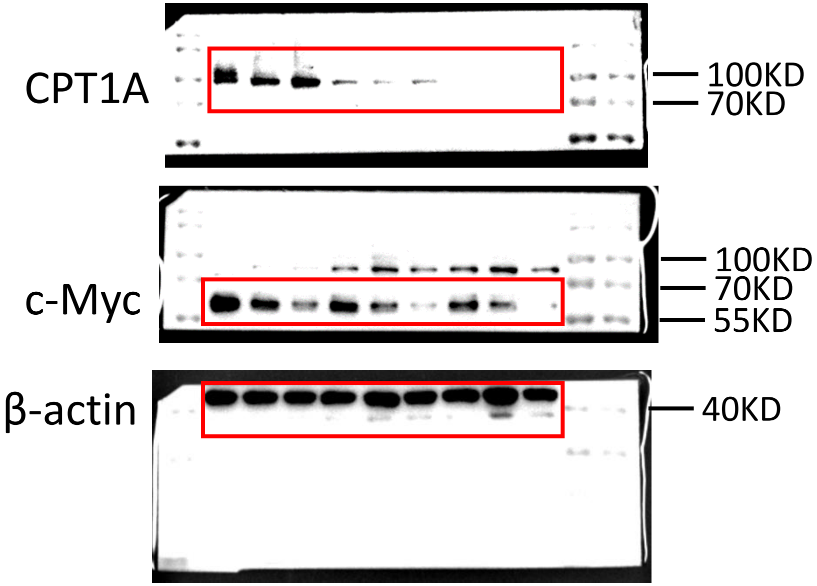
CPT1A
100KD
70KD
c-Myc
100KD
70KD
55KD
β-actin
40KD

Fig.4i

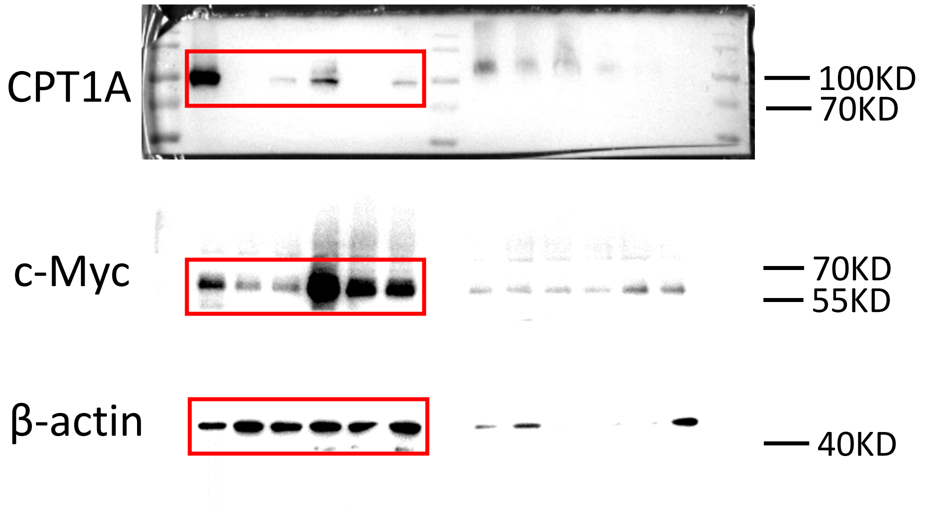

CPT1A
100KD
70KD
c-Myc
70KD
55KD
β-actin
40KD

Fig.4j

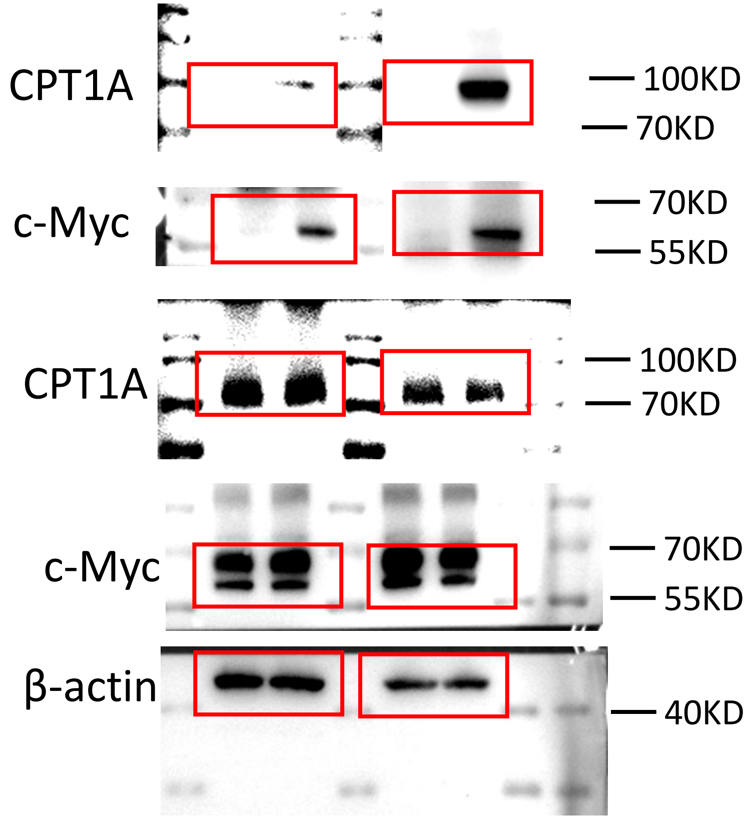

CPT1A
100KD
70KD
c-Myc
70KD
55KD
CPT1A
100KD
70KD
c-Myc
70KD
55KD
β-actin
40KD

Fig.4k

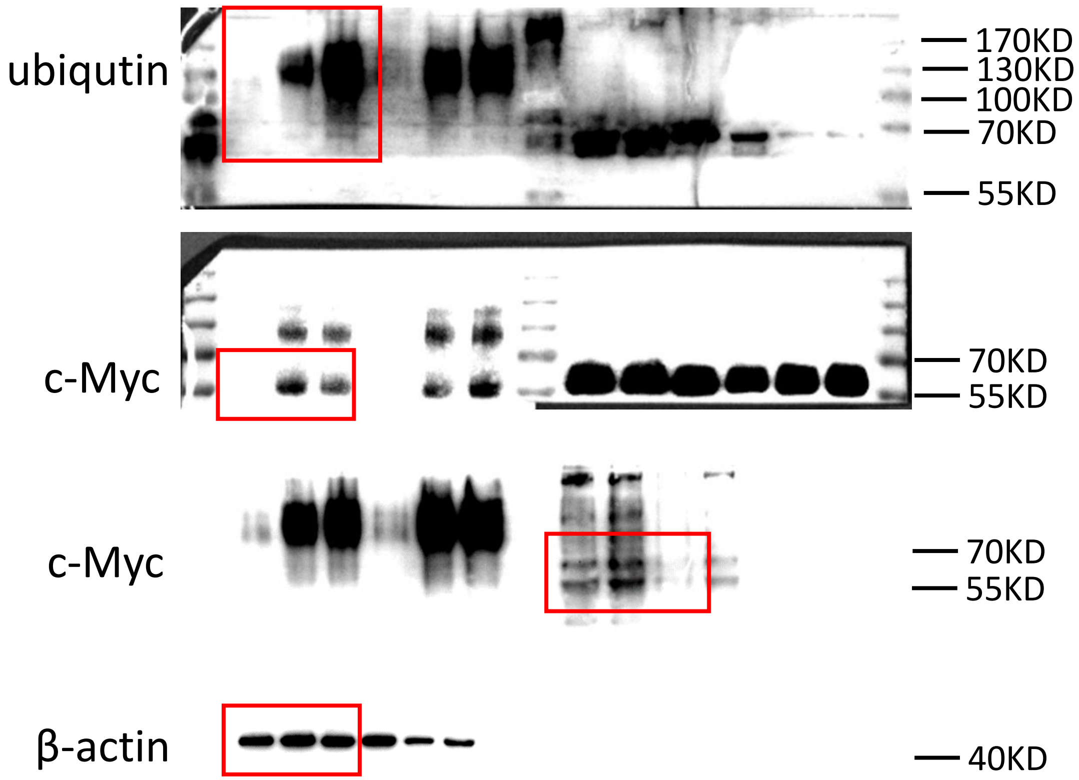
ubiqutin
170KD
130KD
100KD
70KD
55KD
c-Myc
70KD
55KD
c-Myc
70KD
55KD
β-actin
40KD

Fig.4l

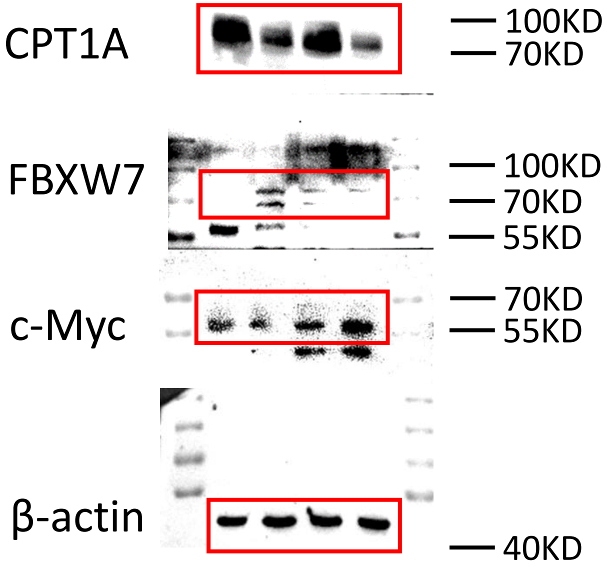
CPT1A
100KD
70KD
FBXW7
100KD
70KD
55KD
c-Myc
70KD
55KD
β-actin
40KD

Fig.4m

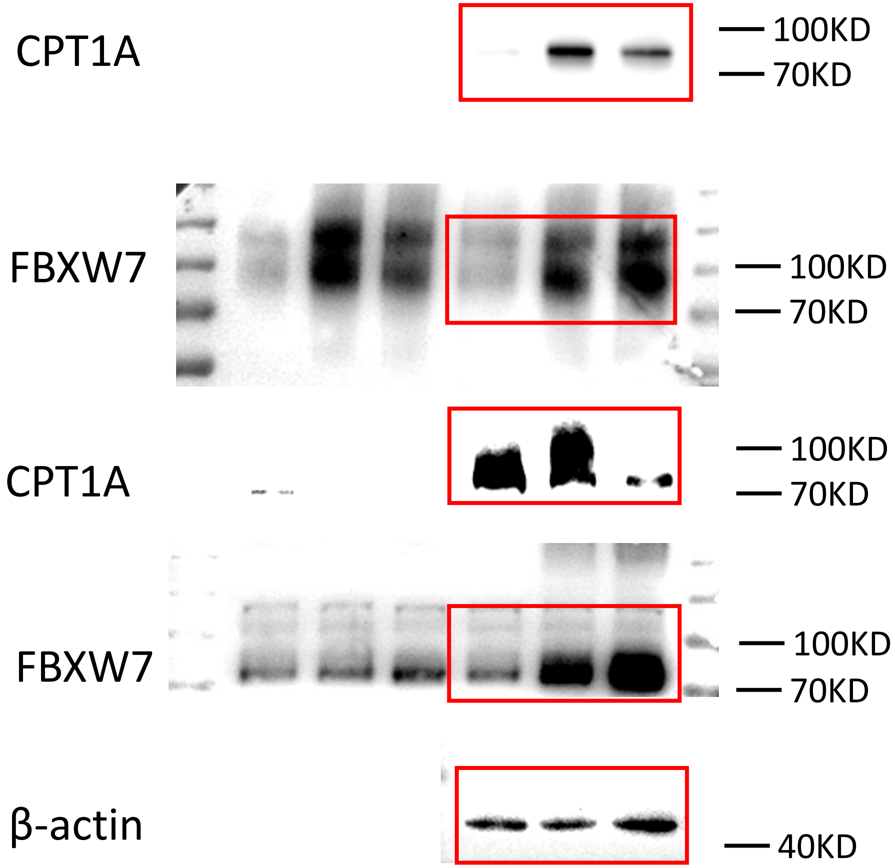

CPT1A
100KD
70KD
FBXW7
100KD
70KD
CPT1A
100KD
70KD
FBXW7
100KD
70KD
β-actin
40KD

Fig.5a

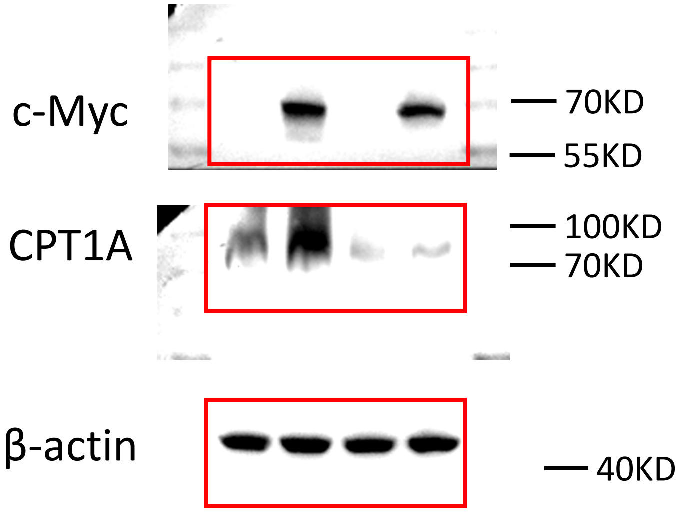

c-Myc
70KD
55KD
CPT1A
100KD
70KD
β-actin
40KD

Fig.5f

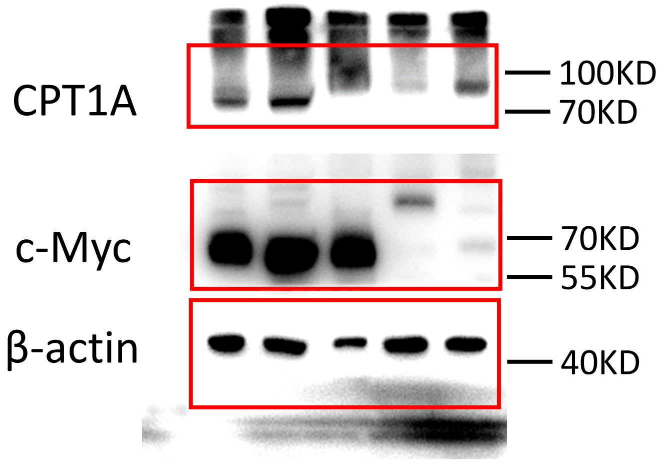
CPT1A
100KD
70KD
c-Myc
70KD
55KD
β-actin
40KD

Fig.6j

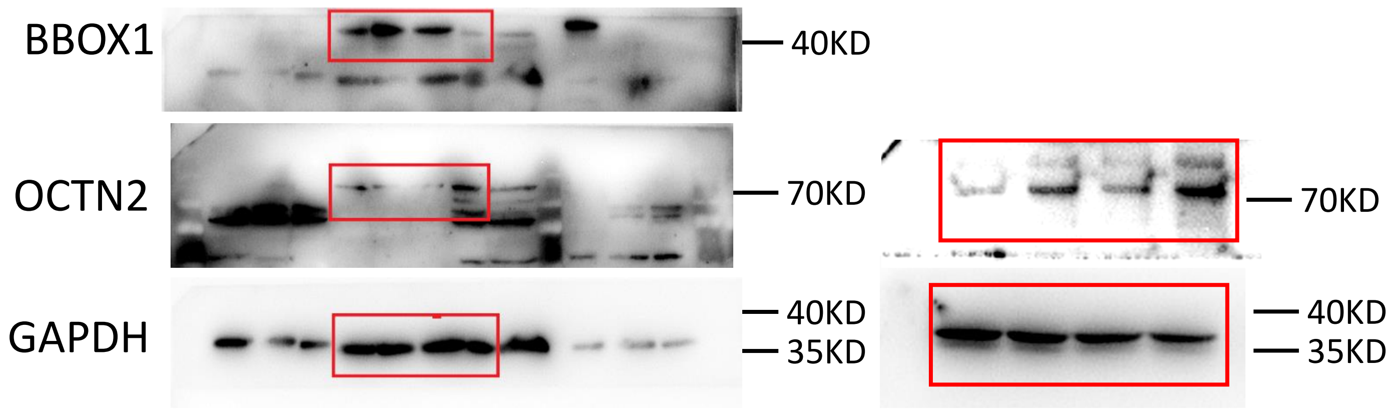
BBOX1
40KD
OCTN2
70KD
70KD
GAPDH
40KD
35KD
40KD
35KD

Fig.S1f

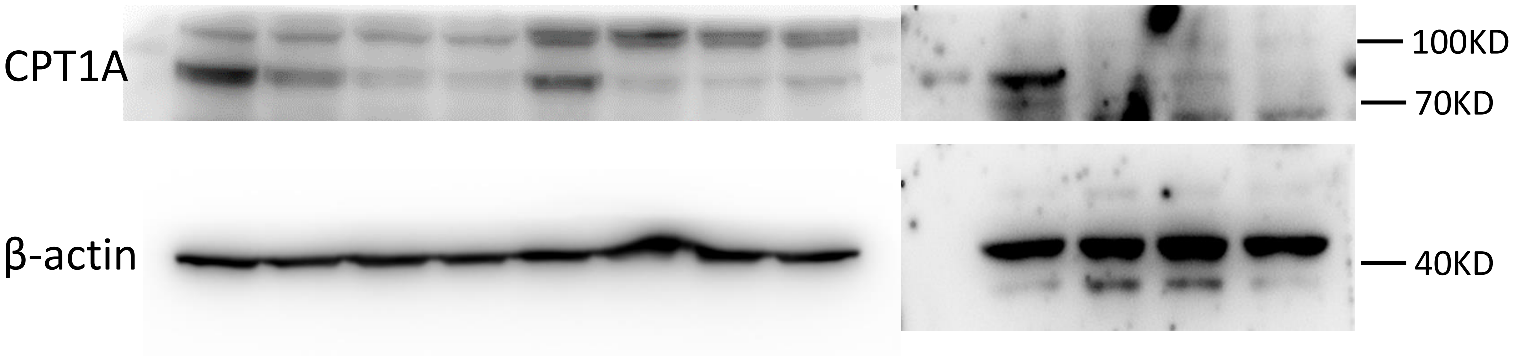

Fig. S1k

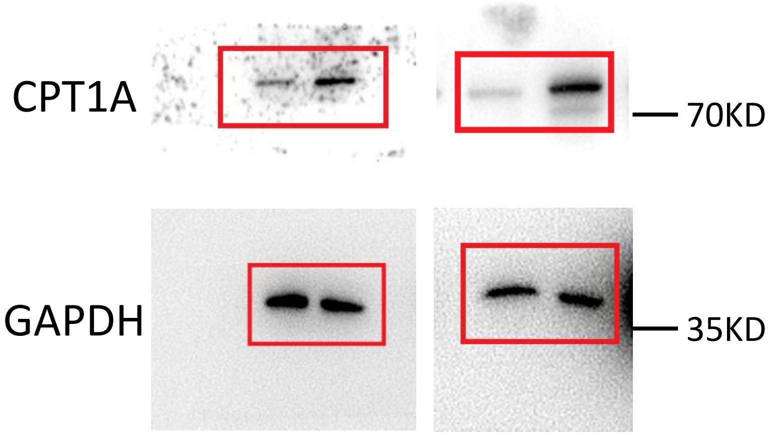

Fig.S3d

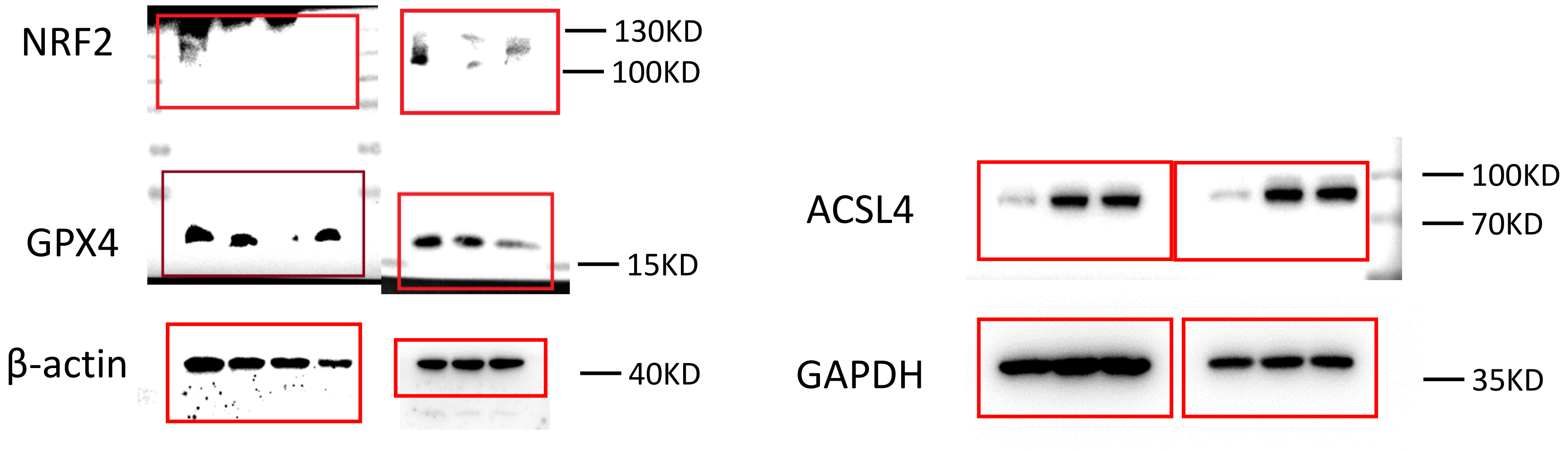
NRF2
130KD
100KD
GPX4
15KD
β-actin
40KD
ACSL4
100KD
70KD
GAPDH
35KD

Fig. S3e

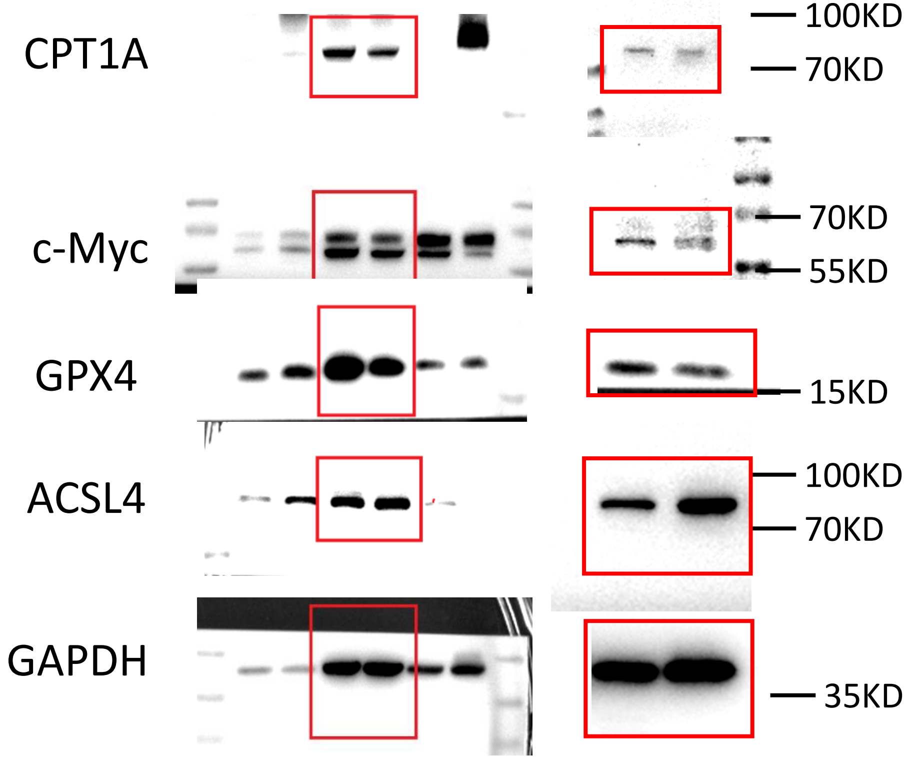

Fig. S4a

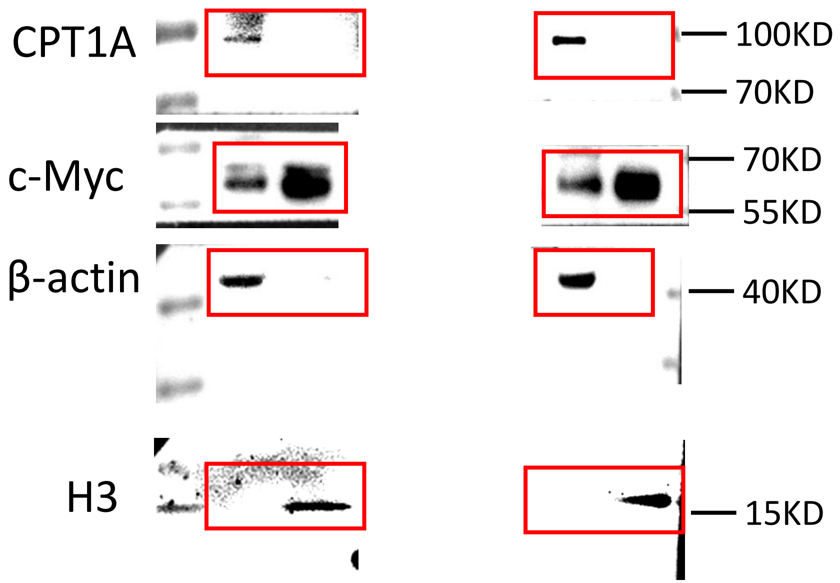

Fig. S4c

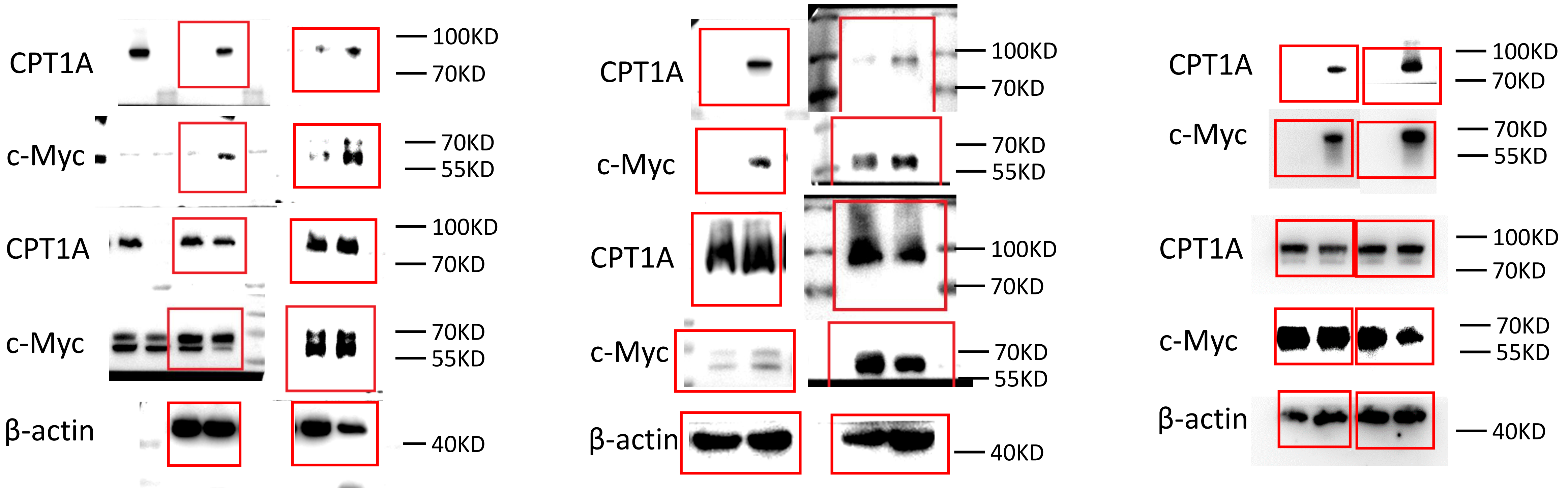

CPT1A
100KD
70KD
c-Myc
70KD
55KD
CPT1A
100KD
70KD
c-Myc
70KD
55KD
β-actin
40KD
CPT1A
100KD
70KD
c-Myc
70KD
55KD
CPT1A
100KD
70KD
c-Myc
70KD
55KD
β-actin
40KD
CPT1A
100KD
70KD
c-Myc
70KD
55KD
CPT1A
100KD
70KD
c-Myc
70KD
55KD
β-actin
40KD

Fig. S4d

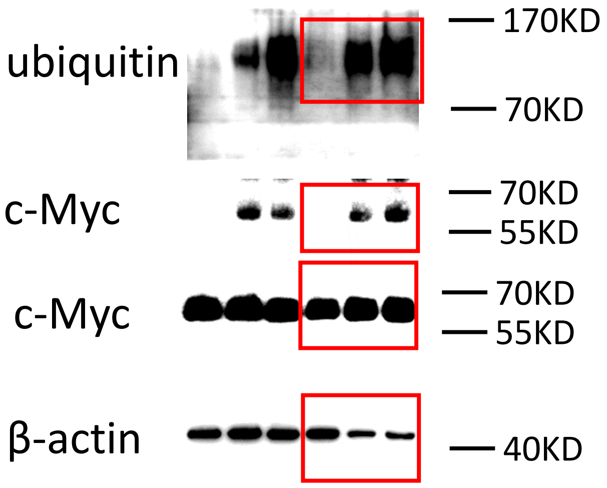

Fig. S6b

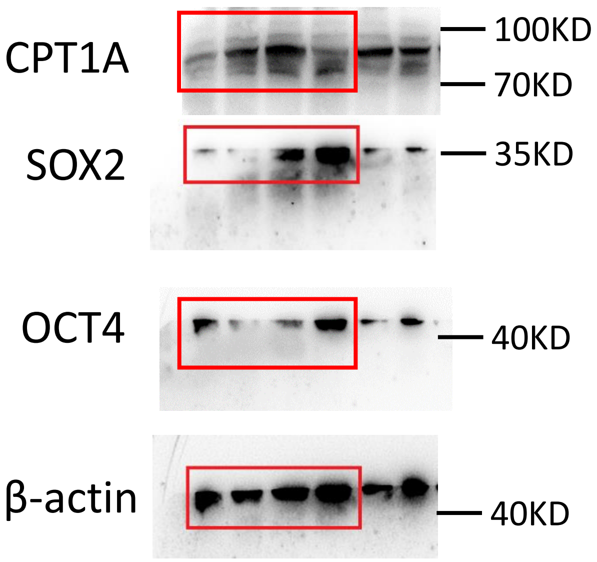

Fig. S6l

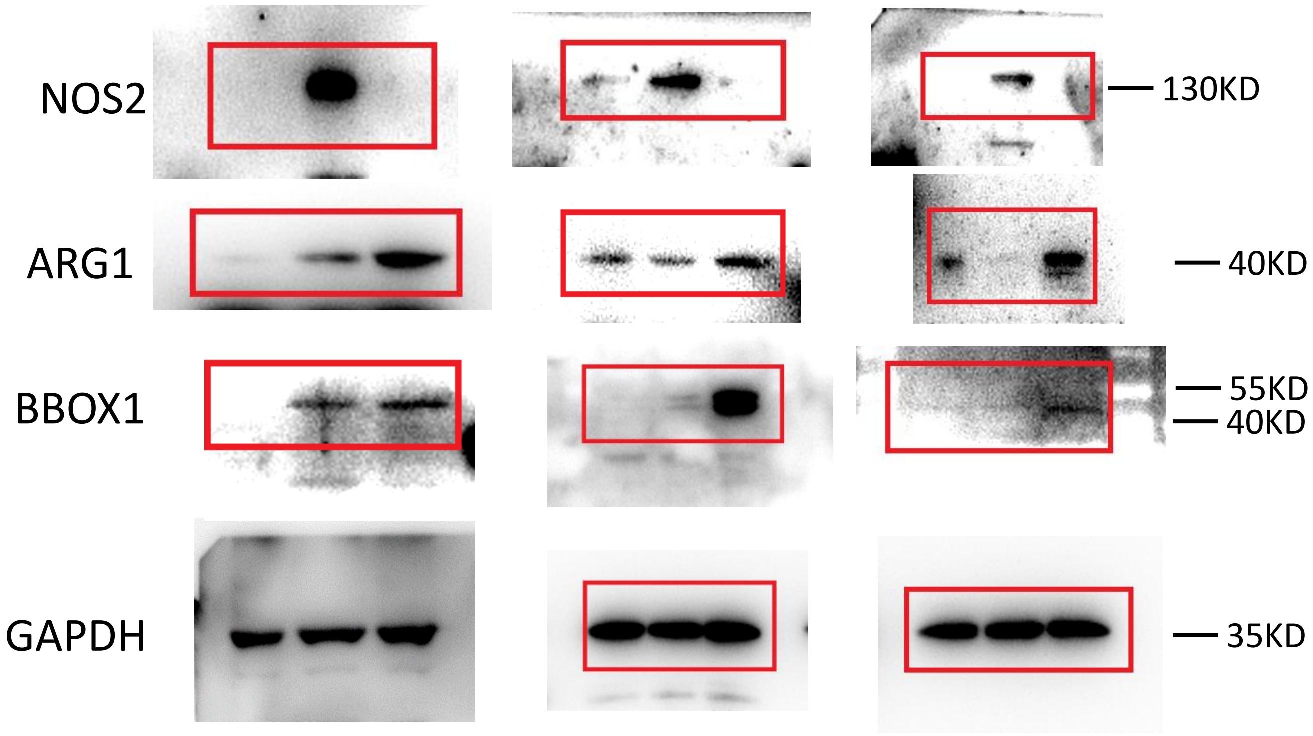

Supplement: Supplementary file 2 — original and uncropped films of Western blots [file 41392_2024_1772_MOESM2_ESM.pdf]
